# The Mediator Subunit, Med23 Is Required for Embryonic Survival and Regulation of Canonical WNT Signaling During Cranial Ganglia Development

**DOI:** 10.3389/fphys.2020.531933

**Published:** 2020-10-22

**Authors:** Soma Dash, Shachi Bhatt, Lisa L. Sandell, Christopher W. Seidel, Youngwook Ahn, Robb E. Krumlauf, Paul A. Trainor

**Affiliations:** ^1^Stowers Institute for Medical Research, Kansas City, MO, United States; ^2^Department of Anatomy and Cell Biology, University of Kansas Medical Center, Kansas City, KS, United States; ^3^Department of Oral Immunology and Infectious Diseases, School of Dentistry, University of Louisville, Louisville, KY, United States

**Keywords:** MED23, Wnt signaling, cranial placodes, cranial ganglia, neural crest cells

## Abstract

Development of the vertebrate head is a complex and dynamic process, which requires integration of all three germ layers and their derivatives. Of special importance are ectoderm-derived cells that form the cranial placodes, which then differentiate into the cranial ganglia and sensory organs. Critical to a fully functioning head, defects in cranial placode and sensory organ development can result in congenital craniofacial anomalies. In a forward genetic screen aimed at identifying novel regulators of craniofacial development, we discovered an embryonically lethal mouse mutant, *snouty*, which exhibits malformation of the facial prominences, cranial nerves and vasculature. The *snouty* mutation was mapped to a single nucleotide change in a ubiquitously expressed gene, *Med23*, which encodes a subunit of the global transcription co-factor complex, Mediator. Phenotypic analyses revealed that the craniofacial anomalies, particularly of the cranial ganglia, were caused by a failure in the proper specification of cranial placode neuronal precursors. Molecular analyses determined that defects in cranial placode neuronal differentiation in *Med23*^sn/sn^ mutants were associated with elevated WNT/β-catenin signaling, which can be partially rescued through combined *Lrp6* and *Wise* loss-of-function. Our work therefore reveals a surprisingly tissue specific role for the ubiquitously expressed mediator complex protein Med23 in placode differentiation during cranial ganglia development. This highlights the importance of coupling general transcription to the regulation of WNT signaling during embryogenesis.

## Introduction

1% of all live human births exhibit a developmental anomaly, and of those, about one-third affect the head and face. Although jaw and skull anomalies are the most common, defects in cranial nerve development also frequently occur, underscoring their importance for development and survival. Mammals have 12 cranial nerves that control various functions, including jaw movement for talking and eating, facial expression, gathering and transmitting information through the sense organs, as well as regulating heart rate and gut peristalsis. The cranial nerves and their associated ganglia are typically composed of two ectodermally-derived cell populations, neural crest cells and cranial placode cells, which collectively give rise to neurons and glia (D’Amico-Martel and Noden, 1983).

Neural crest cells comprise a migratory stem and progenitor cell population and are derived from the neural ectoderm. Neural crest cells contribute to a diverse array of cells and tissues throughout vertebrate embryos, including much of the bone, cartilage and connective tissue of the craniofacial skeleton, smooth muscle cells and pericytes of the vasculature, melanocytes or pigment cells in the skin, as well as neurons and glia of the peripheral nervous system (Crane and Trainor, 2006; Sandell and Trainor, 2006; Bhatt et al., 2013; Trainor, 2016). Meanwhile, the cranial placodes originate from a single pre-placodal region (PPR) of non-neural ectoderm, which then segregates into individual cranial placode territories as embryogenesis progresses (Baker and Bronner-Fraser, 2001). Some of the placodes are neurogenic and differentiate into cranial nerves, while others as their names suggest differentiate into the paired olfactory, optic and otic sense organs, or the hormone secreting anterior pituitary gland (Harvard Stem Cell Institute, 2008; Saint-Jeannet and Moody, 2014).

To identify, in an unbiased manner, novel genes that play important roles in neural crest, placode and craniofacial development, we performed a three generation forward genetic screen in mice via *N*-ethyl-*N*-nitrosourea (ENU) induced mutagenesis (Sandell et al., 2011). From this screen we identified a mouse mutant, which was termed *snouty*, due to the hypoplastic malformed shape of its frontonasal tissues. Subsequently, we identified a point mutation in *Med23* underlying the etiology of the *snouty* phenotype. *Med23* encodes a subunit of Mediator, which is essential for general and activated transcription (Allen and Taatjes, 2015). In mammals, the Mediator complex consists of about 30 subunits, which are arranged into four modules, Head, Middle, Tail and Kinase, and Med23 is a member of the Tail module (Conaway et al., 2005). These subunits interact with components of the RNA Polymerase II complex as well as diverse transcription factors, thereby coordinating development and cell fate determination (Risley et al., 2010; Li et al., 2015; Ranjan and Ansari, 2018).

In this study, we show that Med23 is essential for embryo survival, and plays a novel critical role in cranial ganglia development. Med23 regulates canonical WNT signaling during cranial placode development, the perturbation of which results in defects in cranial ganglia neuronal differentiation. Consistent with this model, we demonstrate that genetically modulating WNT signaling in *snouty* embryos can ameliorate the neuronal defects in cranial ganglia development. Our work therefore has uncovered an important link between the Mediator complex and WNT signaling, which links the general transcription co-factor machinery to modulation of a major highly conserved signaling pathway important in development, tissue homeostasis and disease.

## Materials and Methods

### Animals

The *snouty* mouse line (*Med23*^sn^) used in this study was generated as previously described (Sandell et al., 2011). DNA from *snouty* mutants and founders was collected and subjected to mapping with microsatellites polymorphic between the mixed C57/BL6 and 129/Sv background of our mutagenized animals and the FVB strain to which they were bred (Sandell et al., 2007, 2011). The *snouty* mutation was initially mapped to proximal region of chromosome 10 and then through single nucleotide polymorphism refinement, was identified as a single T-A base pair change in exon 22 of *Med23*. Genotyping was performed by Transnetyx with the following Taqman probes:

Forward Primer: CAACGACATGGTGTGGAAGTACA
Reverse Primer: TCTTACCAGGCAGAGAATGAGTCT
Reporter 1: CCAGCGTGACAATGT (mutant)
Reporter 2: TCCAGCGTGTCAATGT (wild-type)

The *Med23^bgeo/+^* and *Med23^flox/+^* allele mice were derived from ES cells generated through the Knockout Mouse Project (KOMP) consortium. These mice and their derivatives were genotyped via Transnetyx. *Cre-ER^T2^* [B6.129 – *Gt(ROSA)26Sor^TM 1(Cre–ERT2)Tyj^/J*, Jax stock cat# 008463], *Tek-Cre* [B6.Cg-Tg(Tek-cre)1Ywa/J, Jax Stock cat# 008863], and *Wnt1-Cre* [*H2afv^Tg(Wnt1–cre)11Rth^* Tg(Wnt1-GAL4)11Rth/J, Jax stock cat# 003829] mice were obtained from the Jackson Laboratory and maintained as previously described (Chai et al., 2000; Jiang et al., 2000; Kisanuki et al., 2001). *Med23^flox/flox^* mice were crossed with Cre driver mice in order to delete *Med23* in neural crest cells (*Wnt1-Cre*), in endothelial cells (*Tek-Cre*) or in all cells following treatment with 2 mg of tamoxifen and 0.4 mg progesterone (*Cre-ER^T2^*). *Lrp6*^+/–^ and *Wise*^+/–^ mice were generated and maintained as previously described (Ahn et al., 2010, 2013). For embryonic staging, the morning of identification of the vaginal plug was defined as embryonic day (E)0.5. All procedures were performed in compliance with Stowers Institute of Medical Research (SIMR) IACUC approved protocols (2019-094 and 2019-097).

### Western Blotting

Total protein was extracted from three control and three mutant embryos. Protein concentration was estimated by a BCA assay. A standard western blot was performed using the following primary antibodies: Med23 at 1:500 (LifeSpan BioSciences, cat# LS-C193128) and a-Tubulin at 1:1000 (Life Technologies, cat# MA1-19162).

### Whole Mount Immunostaining

**FIGURE 1:**
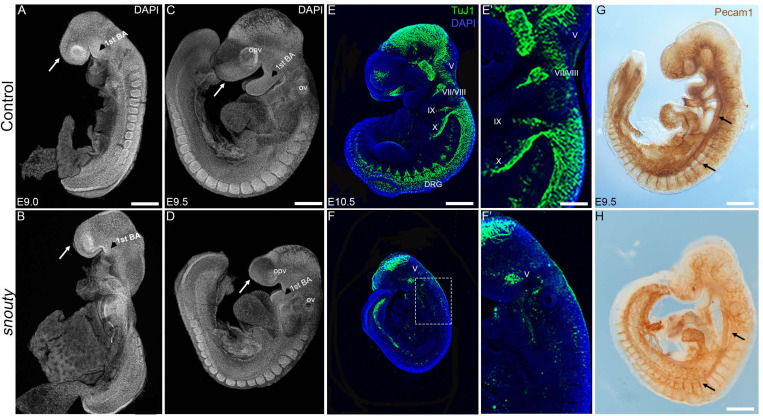
*snouty* embryos exhibit small body size and craniofacial defects at E9.5. **(A–D)** Control and *snouty* mouse embryo littermates at E9.0 and E9.5 were stained with DAPI and imaged with a confocal microscope demonstrating the smaller body size and shortened frontonasal prominences (arrows) in *snouty* embryos. **(E–F′)** Neuronal marker TuJ1 staining of control and *snouty* embryos at E9.5 suggests that *snouty* embryos exhibit disrupted cranial ganglia. **(E′,F′)** Are higher magnification images of **(E,F)**. **(G,H)** PECAM1 immunostaining of E9.5 control and *snouty* embryos reveal abnormal development of the vascular tree like network in the head and epibranchial regions of *snouty* mutants. Intersomitic vessel formation (arrow, **G,H**) is not affected.opv, optic vesicle; ov, otic capsules; BA, branchial arch; DRG, dorsal root ganglia. Scale bars for **(A–D)** is 300 um, **(E,F)** is 350 um, **(G)** is 300 um, **(H)** is 200 um.

Embryos were harvested in 1X PBS and fixed in 4%PFA in PBS at 4°C overnight. Standard whole embryo immunostaining was performed as previously described (Inman et al., 2013; Sandell et al., 2014). Primary antibodies against the following proteins were used: β-tubulin III (TuJ1) at 1:1000 dilution (Covance Research products, cat# MMS-435P), PECAM1/CD31 at 1:400 dilution (BD Pharminogen, cat# 553370), cleaved caspase 3 at 1:1000 dilution (Cell Signaling Technology, cat# 9661S) and Sox10 at 1:500 dilution (Abcam, cat# 155279). Secondary antibodies used were: Goat anti-Mouse IgM (Invitrogen/Molecular Probes, cat# A-21042), Goat anti-Rabbit IgM (Invitrogen/Molecular Probes, cat# A-11034) and Peroxidase AffiniPure Donkey Anti-Rat IgG (H + L) (Jackson ImmunoResearch, cat# 712-035-153) at 1:500 dilution. Immunostained embryos were mounted in Vectashield Antifade Mounting Media (Vectorlabs, cat# H-1000) and imaged using an upright confocal microscope. Except for Pecam1 staining in Figure 1, control and mutant embryos were imaged with the same magnification.

### X-Gal Staining

For whole mount X-Gal staining, the embryos were dissected in Tyrode’s solution and fixed in 2% PFA/0.2% Glutaraldehyde in PBS on ice for varying time periods depending on their embryonic stage (E7.5–15, E8.5–30, E9.5–45 min). Following fixation, the embryos were stained using the X-gal staining kit from Millipore corporation, cat# BG-6-B according to the manufacturer’s protocol.

### RNA *in situ* Hybridization

The embryos were harvested in 1X PBS/0.1% DEPC and fixed in 4% PFA in 1X PBS/0.1% DEPC overnight at 4°C. *In Situ* hybridization was performed using a standard protocol as previously described (Behringer et al., 2014). Control and mutant embryos were imaged at the same magnification using a Nikon camera. Images were processed using Adobe Photoshop.

### Microarray

Mouse Exonic Evidence Based Oligonucleotide (MEEBO) arrays^1^ were printed on poly-L-lysine coated slides using a High-Speed Linear Servo Arrayer. The concentration of oligonucleotides was 40 μM in 3X SSC buffer. Following printing, slides were blocked with succinic anhydride. Microarrays were performed on total RNA isolated from individual E9.5 wild-type and *snouty* mutant littermate embryos using the RNeasy Mini Kit (Qiagen). Aliquots of total RNA were tested for integrity and concentration using the RNA 6000 Nano Assay and RNA LabChips on the Agilent Bioanalyzer 2100. ArrayControl RNA Spikes (Thermo Fisher, cat# AM1780) were mixed with 1 μg of high integrity total RNA before undergoing amplification using the AminoAllyl MessageAmp II aRNA Amplification Kit (Thermo Fisher, cat# AM1753) according to the corresponding instruction manual.

Labeling and hybridization of aRNA was carried out essentially as described previously but with a few modifications (Bozdech et al., 2003). Briefly, 3 μg amplified aRNA was coupled with Cy dyes (CyDye Post-Labeling Reactive Dye Pack, GE Healthcare, RPN 5661). Labeling reactions were quenched with hydroxylamine, and uncoupled dye material removed using cleanup columns following the AminoAllyl MessageAmp II aRNA Amplification Kit instructions (Thermo Fisher, cat# AM1753). Fragmentation of aRNA to between 60 and 200 nt was conducted using (Ambion) RNA Fragmentation Reagents (Thermo Fisher, cat# AM8740) according to the manufacturer’s instructions. Slides were hybridized with a mixture of Cy3 and Cy5 labeled probes. Hybridizations were performed at 63°C overnight under standard conditions (3X SSC, 0.2 mg/ml Poly-dA15, 25 mM HEPES, 0.25% SDS). Slides were then washed at room temperature successively with 0.6X SSC/0.03% SDS and then 0.06X SSC prior to scanning.

Microarray images were acquired with a GenePix 4000B scanner (Axon Instruments, Foster City, CA, United States). Image analysis was performed using GenePix Pro 6.0 software (Axon Instruments). Differentially expressed genes were identified at a significant *p*-value < 0.05 and fold change cut-off of ± 1.2. Microarray data has been deposited in NCBI’s Gene Expression Omnibus and is available through GEO Series accession number GSE144327^2^. Pathway analysis was performed using Ingenuity Pathway Analysis (IPA®). Cytoscape was used to perform network analyses.

### qRT-PCR

Total RNA was isolated from the heads of individual E9.5 wild-type, *Med23*^sn/sn^, *Med23*^sn/sn^;*Lrp6^+/–^, Med23*^sn/sn^;*Wise*^+/–^ and *Med23*^sn/sn^;*Lrp6*^+/–^;*Wise*^+/–^ embryos using the RNeasy Mini Plus Kit (Qiagen). cDNA synthesis and RT-qPCR were performed as described on an ABI7300 Real-Time PCR system using Power Sybr Green PCR master mix (Invitrogen Life Technologies, Carlsbad, CA, United States). For each sample, differential expression was determined using the ΔΔCT method and a *t*-test was used for statistical analysis. The following primers were used for qRT-PCR: *Med23* Forward: 5′-ATTACAAGGGTGTTCGAGA-3′; *Med23* Reverse 5′-TATAACCTCTCTTGCTGCCAGA-3′; *Dkk1* Forward: 5′-CA ACTACCAGCCCTACCCTTG-3′, *Dkk1* Reverse: 5′-GAGCCT TCTTGTCCTTTGGTGTGA-3′, *Ccnd1* Forward: 5′-TGGATG CTGGAGGTCTGTG-3′, *Ccnd1* Reverse: 5′-ACTTCACATCTG TGGCA-3′, *Ngn1* Forward: 5′-CAAGCCCATTCACTCCCTGA-3′, *Ngn1* Reverse: 5′-CAAGCCCATTCACTCCCTGA-3′, *Ngn2* Forward: 5′-GAGCCGCGTAGGATGTTCGTCA-3′, *Ngn2* Reverse: 5′-CCTGCCCGGCTTCCGCTCCA-3′, *NeuroD1* Forward: 5′-GCTGTTTGAGATGTGATGCTGG-3′, and *NeuroD1* Reverse: 5′-AGACGTTGATCCTCCTCGCT-3′. Primers to detect *Med23* transcripts were designed based on the exon–exon junction of exons 1–2 and exons 3–4 of the *Med23* gene.

## Results

### *snouty* Embryos Exhibit Defects in Craniofacial, Cranial Ganglia, and Vascular Development

We identified 10 distinct recessive mutants in our ENU mutagenesis screen, each of which presented with craniofacial dysmorphogenesis at E9.5-E10.5 (Sandell et al., 2011). One mutant was termed *snouty* due to the characteristic hypoplasia of its frontonasal prominence and pharyngeal arches, which was evident as early as E9.0 (Figures 1A,B). By E9.5, *snouty* embryos exhibit defects in overall growth compared to littermate controls with progressively worsening craniofacial abnormalities, including malformed frontonasal prominences, pharyngeal arches, otic and optic vesicles (Figures 1C,D). Relative to control littermates, *snouty* embryos are also slightly developmentally delayed at this stage, by about 4 h, as measured by their difference in somite number. *snouty* embryos are embryonic lethal around E10.5.

The nervous and vascular systems are two precisely patterned networks that develop in close proximity to each other during embryogenesis. Perturbation of either system or their integration can lead to neurovascular disorders, which are a major cause of embryonic lethality (Shalaby et al., 1995; Lim et al., 2000, 3; Ruhrberg and Bautch, 2013). Therefore, as a first step toward understanding the mechanistic origin of the craniofacial defects and the lethality observed in *snouty* embryos, we examined whether neurovascular development occurred normally in *snouty* embryos. Using β-tubulin III (TuJ1) which labels developing neurons and their axons in the central and peripheral nervous systems (CNS and PNS), we observed that a complex neuronal network forms in the midbrain (CNS) of wild-type embryos by E10.5 (Figures 1E,E′). Furthermore, β-tubulin III demarcates developing neurons of the cranial sensory nervous system, including the trigeminal (V), facial/vestibulo-acoustic (VII/VIII), glossopharyngeal (IX) and vagal (X) nerves, as well as dorsal root ganglia (DRG) in the trunk. In contrast, in *snouty* mutant embryos, although a midbrain neuronal plexus is formed, β-tubulin III immunostaining revealed that the network was hypoplastic, and furthermore that there was a paucity of neurons in the cranial sensory ganglia, with respect to the trigeminal, facial, and glossopharyngeal nerves (Figures 1F,F′). Thus, *snouty* embryos exhibit major perturbations in nervous system development, particularly the PNS when compared to control littermates, and this may underpin their early embryonic lethality (Figures 1E,F).

Defective vascular development is also a frequent cause of embryonic lethality and platelet endothelial cell adhesion molecule (PECAM1/CD31), which labels endothelial cells amongst others, illustrates the complexity of the developing vascular network during embryogenesis (Figures 1G,H). Similar to wild-type embryos, PECAM1 immunostaining revealed a well-developed network of embryonic blood vessels in *snouty* mutant embryos. This is evident in the dorsal aorta and intersomitic vessels. However, the complexity and density of the vessel pattern, particularly in the midbrain and pharyngeal arch arteries were noticeably reduced (Figures 1G,H). Perturbation of the vascular network was not perceived to be as severe as that of the neural network, suggesting the gene mutated in *snouty* embryos played a predominant role in regulating cranial sensory nervous system development.

### *snouty* Carries a Single Point Mutation in *Med23*, Which Encodes a Ubiquitously Expressed Subunit of the Mediator Complex and Is Essential for Embryo Survival

**FIGURE 2:**
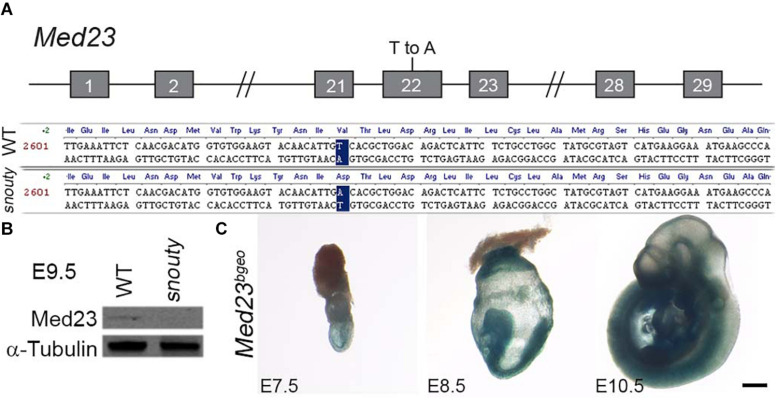
s*nouty* mutant mice have a mutation in the *Med23* gene. **(A)** The *snouty* mouse mutant phenotype was determined to be caused by a single base pair mutation in Exon 22 of the *Med23* gene, which leads to a valine to aspartic acid amino acid change in the protein. Chromosome position of the mutation is noted based on mm10 mouse assembly. **(B)** Western blot against Med23 shows a drastic reduction of Med23 protein levels in *snouty* mutant embryos. **(C)** Ubiquitous expression of LacZ throughout E7.5 to E10.5 embryos is indicative of the Med23 expression. Scale bar is 250 um.

To identify the DNA mutation associated with the *snouty* phenotype, we used a panel of microsatellite markers polymorphic between the mixed C57/BL6 and 129/Sv background of our mutagenized animals and the FVB strain to which they were bred (Sandell et al., 2007, 2011). The *snouty* mutation was initially mapped to chromosome 10 and then through single nucleotide polymorphism refinement, was identified as a single T-A base pair change in exon 22 of *Med23* (Figure 2A). *Med23* encodes a subunit of the transcription co-factor complex, Mediator. The missense base pair change which maps to the core of Med23, led to a single amino acid change from Valine to Aspartic acid (V to D) in the 6-HEAT motif that contributes to the arch-shaped structure or conformation of Med23 (Monté et al., 2018). Western blotting indicated that Med23 is essentially absent in *snouty* mutant embryos (Figure 2B). The reduced protein level is consistent with a potential conformational change rendering the mutant Med23 protein unstable.

To definitively prove that *Med23* loss-of-function was responsible for the *snouty* phenotype, we performed a complementation test with a null allele of *Med23* generated from ES cells obtained through the Knock-out Mouse Project (KOMP) consortium. The ES cells contained a splice acceptor-lacZ genetrap construct inserted between exons 12 and 13 of *Med23* (Supplementary Figure S1A). The 5′ splice acceptor site and 3′ polyA tail were designed to produce a null allele by disrupting the endogenous *Med23* transcript through expression of a reporter lacZ fusion transcript. Mice carrying the genetrap allele were designated *Med23*^bgeo/+^. *Med23*^bgeo/bgeo^ homozygous embryos displayed similar developmental defects to *snouty* (hereafter referred to as *Med23*^sn/sn^) embryos and did not survive beyond E10.5 (Supplementary Figures S1B,C). *Med23*^bgeo/bgeo^ embryos were also developmentally delayed relative to control littermates, and perhaps slightly more so than *Med23*^sn/sn^ embryos. This is likely due to the complete loss of function of Med23 in *Med23*^bgeo/bgeo^ embryos as well as the C57BL/6 background of the *Med23*^bgeo/bgeo^ embryos compared to FVB background for *Med23*^sn/sn^ embryos.

For the complementation test, we generated *Med23^sn/bgeo^* embryos and compared their phenotype to *Med23*^sn/sn^ and *Med23*^bgeo/bgeo^ embryos (Supplementary Figure S2). Similar to *Med23*^sn/sn^ and *Med23*^bgeo/bgeo^ embryos, *Med23^sn/bgeo^* embryos also do not survive beyond E10.5, and display craniofacial, neuronal and vascular development defects that are similar to *Med23*^sn/sn^ and *Med23*^bgeo/bgeo^ embryos (Supplementary Figures S2A–F). The failure of the *Med23*^bgeo^ allele to complement the *Med23^sn^* allele provided genetic confirmation that Med23 loss-of-function is responsible for the *Med23*^sn/sn^ phenotype and furthermore that *Med23*^sn^ is likely a null allele similar to *Med23*^bgeo^.

Because *Med23* has been previously identified as a candidate gene for placental defects (Perez-Garcia et al., 2018), we examined the placentas of wild-type and *Med23*^sn/sn^ embryos at E9.5 by histology. The primary layers, trophoblast giant cell layer, chorion and allantois, appear to be present in *Med23*^sn/sn^ placenta and in similar proportions compared to wild-type placenta. This suggest that there are no gross morphological defects in placenta development in *Med23*^sn/sn^ embryos (Supplementary Figure S3). This could be because the *snouty* point mutation in *Med23* does not affect placental development as opposed to the *bgeo* insertional deletion of *Med23*. Another possibility is that genetic background affects placental development (Dackor et al., 2009). *Med23*^sn/sn^ embryos are on an FVB background in contrast to the C57Bl/6N background in which the placental defects were previously described.

Therefore, to begin to understand the mechanistic origins and pathogenesis of the craniofacial, neuronal and vascular defects observed in the allelic series of *Med23* embryos, we characterized the spatiotemporal expression of *Med23* during normal embryogenesis. Using the lacZ fusion transcript as a reporter for Med23 activity, X-gal staining of *Med23*^bgeo/+^ embryos revealed that *Med23* is expressed ubiquitously in all embryonic tissues during post-implantation development from E7.5 to 10.5 (Figure 2C).

### Cranial Ganglia Defects in *Med23*^sn/sn^ Embryos Occur Predominantly Due to Defects in Cranial Placode Development

**FIGURE 3:**
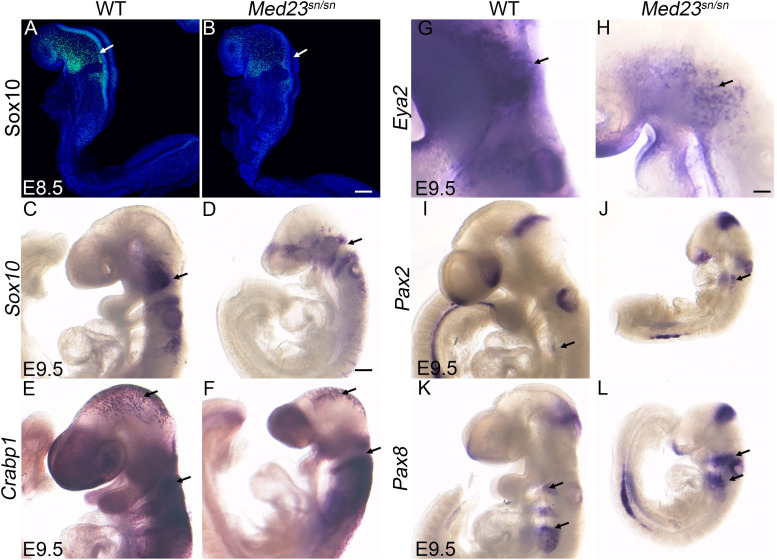
*Med23*^sn/sn^ mutants display neural crest cell and placodal defects. **(A–D)** Sox10 expression in E8.5 and E9.5 control and *Med23*^sn/sn^ embryos shows reduced *Sox10* + cells entering the cranial ganglia region near the 1^st^ branchial arch and around the otic vesicle in *Med23*^sn/sn^ embryos. **(E,F)** *Crabp1* ISH reveals proper neural crest cell migration in the cranial and trunk regions of E9.5 control and *Med23*^sn/sn^ embryos. **(G,H)** *Eya2* expression in control and *Med23*^sn/sn^ embryos shows proper formation of anterior pre-placodal area. However, very few *Eya2*^+^ cells are observed in the trigeminal and epibranchial regions of *Med23*^sn/sn^ embryos (arrow, **H**). **(I,J)** *Pax2*^+^ cells are present in the optic, otic, and isthmus of both control and *Med23*^sn/sn^ embryos. **(K,L)** *Pax8*^+^ cells are present but loosely organized in the epibranchial region, otic placode and isthmus of *Med23*^sn/sn^ embryos in comparison to controls. Scale bar for **(A,B)** is 120 um, **(C–F,I–L)** is 300 um, and **(G–H)** is 150 um.

*Med23*^sn/sn^ embryos are characterized primarily by hypoplastic frontonasal prominences and pharyngeal arches as well as a malformed peripheral nervous system, particularly of the cranial sensory ganglia (Figure 1). Previous studies have shown that the cranial ganglia are derived from two distinct ectodermal cell populations; neural crest cells and placode cells (D’Amico-Martel and Noden, 1983). Cranial neural crest cells contribute neurons and glia, whereas the ectodermal placodes only contribute neurons of the cranial ganglia. However, cellular interactions between neural crest cells and placodes are also essential for proper cranial nerve development (Shiau et al., 2008; Freter et al., 2013; Sandell et al., 2014; Kurosaka et al., 2015). In order to determine whether Med23 was required in neural crest cells and/or cranial placode cells, for proper cranial sensory ganglia development, we analyzed their contributions with cell specific markers via RNA *in situ* hybridization in wild-type and *Med23*^sn/sn^ littermate embryos. *Sox10* labels migrating neural crest cells as they delaminate from the neuroepithelium beginning around E8.25–8.5, and becomes progressively restricted to neural crest cells destined for neuro-gliogenic fates around E9.0–E9.5 (Britsch et al., 2001). *Sox10* expression is observed in neural crest cells populating the cranial ganglia in E8.5–9.5 wild-type and *Med23*^sn/sn^ littermate embryos (Figures 3A–D). However, E9.5 *Med23*^sn/sn^ embryos display a slight reduction in *Sox10*^+^ cells in the head (Figures 3B,D). *Crabp1* labels the neuroepithelium, midbrain neuronal plexus, and migrating neural crest cells that colonize the pharyngeal arches in E9.0–9.5 control embryos (Dencker et al., 1990). Although the levels of *Crabp1* expression are comparable, the domains are somewhat reduced in *Med23*^sn/sn^ mutant embryos (Figures 3E,F). Collectively, the expression of *Sox10* and *Crabp1* in *Med23*^sn/sn^ embryos relative to controls indicates that Med23 is not required for the induction or migration of cranial neural crest cells. Thus, defects in neural crest cell development are unlikely to cause the cranial peripheral nervous system defects observed in *Med23*^sn/sn^ embryos.

Therefore, we turned our attention to cranial placode development, the perturbation of which could underlie the malformed cranial sensory ganglia observed in *Med23*^sn/sn^ embryos (Figure 1). Each stage of cranial sensory development, from specification of the pre-placode territory to neuronal differentiation of cranial placode cells, can be delineated via spatiotemporal gene expression (Schlosser, 2006; Saint-Jeannet and Moody, 2014). Cranial placode development begins with the formation of a single ectodermal-cell population called the pre-placodal region. *Six* and *Eya* gene family members are expressed at the time of pre-placodal region specification and their expression is maintained as the pre-placodal region segregates and differentiates into specific placodes (Zou et al., 2004). At E9.5, *Eya2*^+^ cells are observed in the trigeminal and epibranchial placodes of wild-type embryos (Figure 3G). In contrast, *Med23*^sn/sn^ embryos display a reduced domain of *Eya2*^+^ cells in the developing trigeminal placode and forming epibranchial placodes (Figure 3H). Interestingly, *Eya2^–/–^* mutant mice present with no obvious external phenotype and are viable and fertile (Grifone et al., 2007), indicating that loss of *Eya2* expression cannot explain the cranial ganglia neurogenesis defects in *Med23*^sn/sn^ embryos. This led us to hypothesize that Med23 is specifically required during later stages of cranial sensory nervous system development, namely segregation and/or differentiation.

To further understand the function of Med23 during cranial placode development, we next examined whether the placodes are appropriately specified in *Med23*^sn/sn^ embryos. More specifically, we characterized the expression of *Pax2* and *Pax8*, which have been shown to demarcate the newly specified epibranchial and otic placodes in mouse embryos (Baker and Bronner-Fraser, 2001; Bouchard et al., 2004; Ohyama and Groves, 2004). In E9.5 wild-type embryos, *Pax2*^+^ cells were observed in the nasal, optic and otic placodes, and in the epibranchial region and isthmus (Figure 3I). A similar pattern of *Pax2* expression was also found in *Med23*^sn/sn^ embryos (Figure 3J). *Pax8* is expressed in the epibranchial and otic placodes as well as in the isthmus of E9.5 wild-type mouse embryos (Figure 3K), and this pattern remained largely unchanged in *Med23*^sn/sn^ littermate embryos (Figure 3L), except that *Pax8*^+^ cells in the developing epibranchial placodes do not seem to coalesce as well in the mutant embryos as they do in wild-type controls. Nonetheless, the presence of *Pax2*^+^ and *Pax8*^+^ cells in the epibranchial region suggests that the epibranchial placodes are being specified in *Med23*^sn/sn^ embryos. The persistence of *Eya2*^+^ cells in both wild-type and *Med23*^sn/sn^ embryos, and proper specification of epibranchial placodes (presence of *Pax2*^+^ and *Pax8*^+^ cells in the epibranchial region) in *Med23*^sn/sn^ embryos, suggests that Med23 is required during later stages of cranial placode development, namely segregation and/or differentiation. Collectively, these results imply that malformation of the cranial sensory nervous system in *Med23*^sn/sn^ embryos possibly occurs during the stage of neuronal progenitor cell specification and delamination, which precedes the later differentiation and maturation of the cranial sensory ganglia.

**FIGURE 4:**
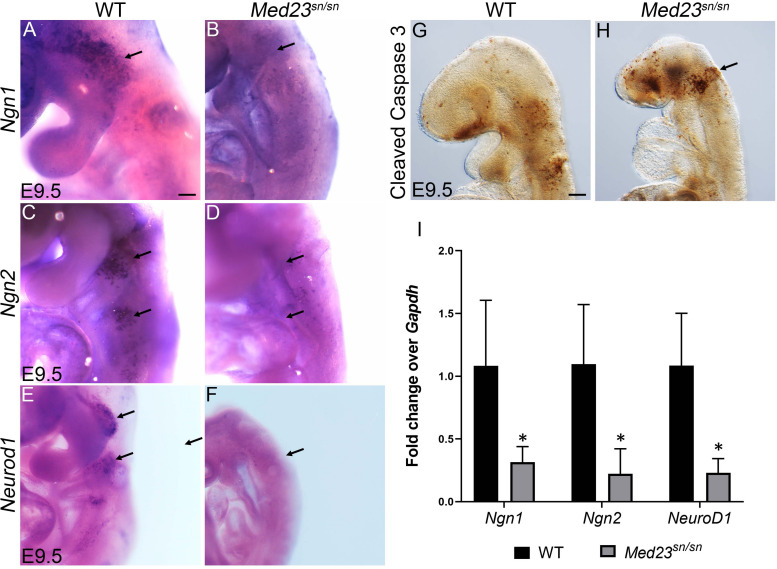
*Med23*^sn/sn^ mutants display defects in neuronal differentiation. **(A,B)** *Ngn1* is expressed in trigeminal (arrow) and epibranchial cells in control embryos, however is downregulated or lost in *Med23*^sn/sn^ embryos. **(C,D)** *Ngn2*^+^ cells are observed in the epibranchial ganglia of control embryos (arrows, **C**). *Ngn2*^+^ cell number is drastically reduced in the epibranchial ganglia of *Med23*^sn/sn^ embryos (arrows, **D**). **(E,F)** *Neurod1*^+^ cells are considerably reduced in the developing trigeminal and epibranchial ganglia of *Med23*^sn/sn^ embryos at E9.5 compared to controls. **(G,H)** A higher number of cleaved Caspase 3 positive cells are observed in the developing trigeminal and frontonasal prominence of E9.5 *Med23*^sn/sn^ embryos compared to controls. **(I)** qPCR analyses of E9.5 wild-type and *Med23*^sn/sn^ littermate embryos revealed downregulation of *Ngn1*, *Ngn2*, and *NeuroD1* transcripts in the mutants. Statistical analysis was performed using ANOVA and ^∗^ denotes a *p*-value < 0.05. Scale bar for **(A–F)** is 150 um, **(G,H)** is 300 um. *p < 0.05.

To delineate the role of Med23 in neuronal specification of cranial placodes cells, we next analyzed the expression of *Neurogenin1* (*Ngn1*) and *Neurogenin2* (*Ngn2*), which are vertebrate neurogenesis determination genes (Fode et al., 1998; Ma et al., 1998). *Ngn1* and *Ngn2* are expressed in the placodal ectoderm prior to neuroblast delamination where they define complementary subsets of cranial sensory neuron precursors. *Ngn1* loss-of-function was previously shown to prevent development of the “proximal” subset of cranial sensory ganglia, whose neurons derive from cranial neural crest, trigeminal, or otic placode precursors (Ma et al., 1998). In contrast, loss of *Ngn2* prevents development of the complementary “distal” subset of ganglia, whose precursors derive from posterior epibranchial placodes (Fode et al., 1998). In E9.5 wild-type embryos, *Ngn1* is primarily expressed in the developing trigeminal placode, but can also be found in the developing epibranchial placodes (Figure 4A). In contrast, very few cells in the developing trigeminal placode of *Med23*^sn/sn^ littermate embryos express *Ngn1*, and no cells in the epibranchial placode region were observed to be *Ngn1*^+^ (Figure 4B). Similar results were observed with respect to *Ngn2*. In E9.5 wild-type embryos, numerous *Ngn2*^+^ cells are present in the epibranchial placodes (Figure 4C), however, in *Med23*^sn/sn^ littermate embryos the region of *Ngn2*^+^ cells in the developing epibranchial placodes was drastically reduced (Figure 4D). We confirmed the downregulation of *Ngn1* and *Ngn2* transcript levels in E9.5 *Med23*^sn/sn^ embryonic heads by qPCR (Figure 4I). These data imply that cranial placode neuronal precursors fail to be specified or delaminate during cranial sensory nervous system development in *Med23*^sn/sn^ embryos.

*Ngn1 and Ngn2* directly regulate *Neurod1* expression in the trigeminal and epibranchial ganglia respectively during cranial sensory nervous system development (Sun et al., 2001; Mattar et al., 2004). *Neurod1*, which is a well-established indicator of neuronal differentiation, is expressed in neurogenic cranial placodal cells in the trigeminal and epibranchial ganglia of wild-type embryos at E9.5 (Figure 4E). *Med23*^sn/sn^ embryos display an almost complete absence of *Neurod1* expression in the developing cranial ganglia (Figure 4F), which we confirmed by qPCR (Figure 4I). The few cells in the trigeminal ganglion that still express *Neurod1* in *Med23*^sn/sn^ embryos correlate with the small number of β-tubulin III^+^ cells observed in the trigeminal ganglion (Figure 1F). In addition, a higher number of cells in *Med23*^sn/sn^ embryos, especially in the trigeminal and the frontonasal prominence are cleaved Caspase 3 positive indicating that cell death is elevated in *Med23*^sn/sn^ embryos compared to controls (Figures 4G,H).

Therefore, our analyses have revealed a requirement for Med23 during multiple steps of cranial placode development, and that a failure in neuronal differentiation and maturation together with apoptosis underlies abnormal development of the cranial sensory nervous system in *Med23*^sn/sn^ embryos.

### Med23 Regulates WNT Signaling During Cranial Ganglia Differentiation

**FIGURE 5:**
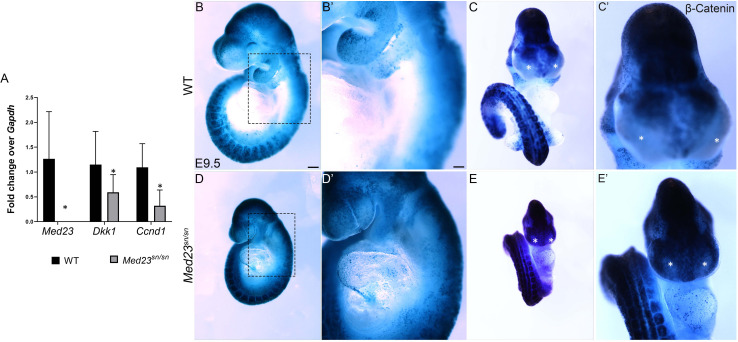
WNT signaling is upregulated and ectopically expressed in the lateral nasal processes of *Med23*^sn/sn^ embryos. **(A)** qPCR analysis of cDNA obtained from E9.5 wild-type and *Med23*^sn/sn^ littermate embryos shows a reduction in the levels of *Med23*, *Dkk1*, and *Ccnd1*. **(B–E′)** Lateral and frontal images of control BATGal and *Med23^sn/sn^;*BATGal embryos reveal an increase in WNT signaling in the frontonasal processes at E9.5. The lateral nasal processes, which are usually devoid of active WNT signaling (*, asterisk, control) exhibit positive LacZ staining (WNT activity) in *Med23*^sn/sn^ embryos (*, asterisk, *Med23*^sn/sn^). **(B′–E′)** are high magnification images of **(B–E)**. Scale bar for **(B–E)** is 450 um, **(B′–E′)** is 100 um.

To determine how Med23 regulates differentiation of the trigeminal and epibranchial placodes during cranial sensory nervous system development, we undertook comparative transcriptome analyses of E9.5 *Med23*^sn/sn^ embryos and their control littermates. Consistent with our spatiotemporal analyses of gene expression, *NeuroD1* was downregulated. More importantly, Ingenuity Pathway Analysis (IPA®) and a Cytoscape network plot revealed that the differential expression of multiple genes in *Med23*^sn/sn^ embryos was associated with the canonical WNT/β-catenin signaling pathway (Supplementary Table S1 and Supplementary Figure S7). Differentially expressed genes include the WNT inhibitor *Dkk1*, and WNT target, *Ccnd1*, both of which were downregulated. We confirmed the downregulation of *Dkk1 and Ccnd1* transcripts in concert with *Med23* loss-of-function using qPCR (Figure 5A), which led us to hypothesize that WNT signaling may be mis-regulated in *Med23*^sn/sn^ embryos. To test this hypothesis, we bred the *BATGal* transgenic reporter into the background of *Med23*^sn/sn^ embryos. *BATGal* reporter mice express *lacZ* under the regulation of β-catenin responsive elements, such that *lacZ* expression serves as a proxy for spatiotemporal WNT signaling (Ahn et al., 2010, 2013). WNT/β-catenin signaling levels, as evidenced by the intensity and spatial distribution of lacZ activity, were increased in the pharyngeal/epibranchial region (dotted box) and lateral frontonasal processes (asterisk) of *Med23*^sn/sn^ embryos, coinciding with the anatomical regions most disrupted (Figures 5B–E′). This suggests that elevated WNT signaling is associated with abnormal morphological development in *Med23*^sn/sn^ embryos.

### Genetic Modulation of WNT Signaling in *Med23*^sn/sn^ Embryos Results in Partial Restoration of Cranial Sensory Neuron Differentiation

WNT/β-catenin signaling is well-known for regulating crucial aspects of mammalian embryonic development (Wodarz and Nusse, 1998), and is associated with ectodermal placode formation (Ahn, 2015). WNT/β-catenin signaling requires interaction between Wnt ligands, Frizzled receptors and WNT co-receptors, Lrp5 and Lrp6 that leads to stabilization and nuclear localization of β-catenin (MacDonald et al., 2009). In contrast, Wise (also known as Sostdc1) modulates WNT/β-catenin signaling by binding to WNT agonists, Lrp5 and Lrp6 and WNT antagonist, Lrp4 (Itasaki et al., 2003; Ahn et al., 2013, 2017). We hypothesized that an up-regulation of WNT/β-catenin signaling was responsible for pathogenesis of the *Med23*^sn/sn^ embryo phenotype and furthermore that genetically modulating WNT signaling might ameliorate the anomalies observed in *Med23*^sn/sn^ mutant embryos. To test this hypothesis, we bred loss-of-function alleles of *Lrp6* and *Wise*, into the background of *Med23*^sn/sn^ embryos. In control experiments, we first confirmed that loss of a single allele of *Lrp6* or *Wise* did not affect craniofacial or neuronal development in E10.5 *Lrp6*^+/–^ and *Wise*^+/–^ embryos (Supplementary Figures S4A–C). Then we ascertained that *Med23*^sn/sn^ embryos do not display any gross aberration in *Lrp6* or *Wise* expression (Supplementary Figures S4D–G).

**FIGURE 6:**
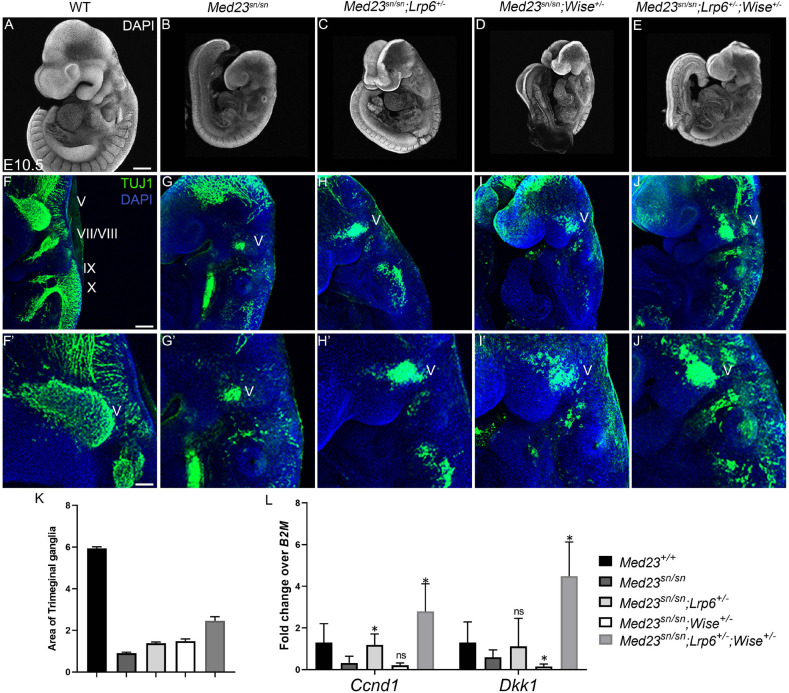
Rescue of the *Med23*^sn/sn^ phenotype by modulating WNT signaling. DAPI stained E9.5 **(A)** wild-type, **(B)** *Med23*^sn/sn^, **(C)** *Med23*^sn/sn^; *Lrp6^+/–^*, **(D)** *Med23*^sn/sn^; *Wise*^+/–^ and **(E)** *Med23*^sn/sn^; *Lrp6*^+/–^; *Wise*^+/–^ embryos. TuJ1 immunostained (green) E9.5 **(F)** wild-type **(G)** *Med23*^sn/sn^ **(H)** *Med23*^sn/sn^; *Lrp6^+/–^*, **(I)** *Med23*^sn/sn^; *Wise*^+/–^ and **(J)** *Med23*^sn/sn^; *Lrp6*^+/–^; *Wise^+/–^*, embryos. Combinatorial loss of *Lrp6* and *Wise* restores neuronal differentiation in the epibranchial region of *Med23*^sn/sn^ embryos. **(F′–J′)** are higher magnification images of **(F–J)** focused on the trigeminal ganglia. **(K)** Quantification of the area of Tuj1 staining in the trigeminal ganglia is shown as a ratio of the length of the embryo for wild-type, *Med23*^sn/sn^, *Med23*^sn/sn^; *Lrp6*^+/–^, *Med23*^sn/sn^; *Wise*^+/–^ and *Med23*^sn/sn^; *Lrp6*^+/–^; *Wise*^+/–^ **(L)** qPCR analysis of cDNA obtained from E9.5 wild-type, *Med23*^sn/sn^, *Med23*^sn/sn^; *Lrp6*^+/–^, *Med23*^sn/sn^; *Wise^+/–^*, and *Med23*^sn/sn^; *Lrp6*^+/–^; *Wise*^+/–^ embryos indicates a significant increase in *Ccnd1* and *Dkk1* transcripts in *Med23*^sn/sn^; *Lrp6*^+/–^; *Wise*^+/–^ embryos compared to WT and *Med23*^sn/sn^. Statistical analysis was performed using Students *t*-test with *Med23*^sn/sn^ as one of the nominal variables. Scale bars for **(A–E)** is 300 um, **(F–J)** is 200 um, **(F′–J′)** is 50 um. *p < 0.05.

Interestingly, although compound *Med23^sn/sn^;Lrp6^+/–^, Med23^sn/sn^;Wise^+/–^* and *Med23^sn/sn^;Lrp6^+/–^;Wise^+/–^* embryos do not exhibit any gross morphological rescue of craniofacial defects or embryonic lethality (Figures 6A–E), the *Med23^sn/sn^; Lrp6^+/–^; Wise^+/–^* compound mutants display a partial restoration of cranial ganglia neurogenesis (Figures 6F–K). More specifically, E9.5 *Med23^sn/sn^; Lrp6^+/–^; Wise^+/–^* embryos exhibit considerable β-tubulin III + staining of the trigeminal (V), facial/vestibulo-acoustic (VII/VIII), glossopharyngeal (IX) and vagal (X) nerves, as well as within the epibranchial region, which is in striking contrast to control, *Med23*^sn/sn^ littermate embryos that show little to no β-tubulin III staining in the equivalent tissues and regions (Figure 6G). This demonstrates that a reduction in the levels of WNT signaling is sufficient to partially rescue some of the defects in cranial placode neuronal differentiation. In addition, we observed a rescue in the expression of *Dkk1 and Ccnd1* transcripts in *Med23^sn/sn^;Lrp6^+/–^;Wise^+/–^* compared to *Med23*^sn/sn^ embryos using qPCR (Figure 6L).

### Med23 Is Not Intrinsically Required in Neural Crest Cells or Endothelial Cells During Cranial Ganglia Development

**FIGURE 7:**
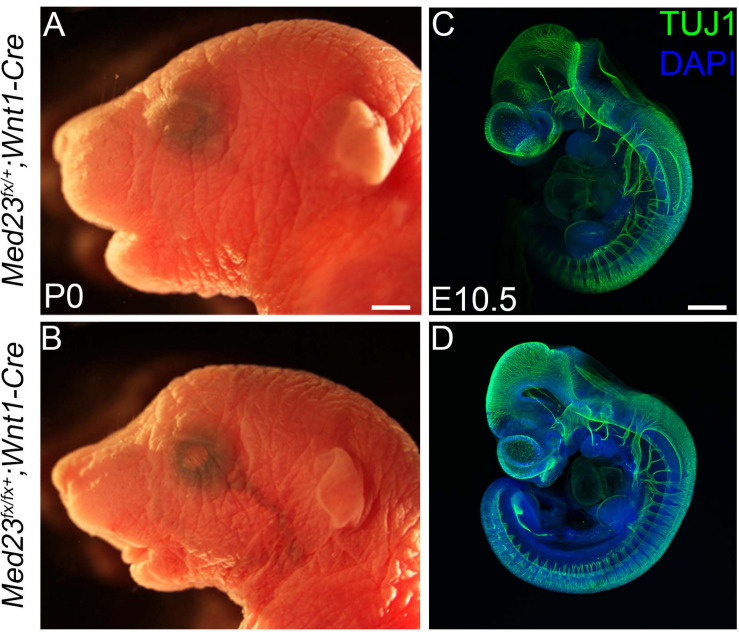
Conditional deletion of *Med23* in neural crest cells. **(A,B)** Brightfield images of P0 *Med23^fx/+^;Wnt1-Cre* and *Med23^fx/fx^;Wnt1-Cre* embryos indicate that the mutants exhibit micrognathia. **(C,D)** TuJ1 staining of E10.5 *Med23^fx/+^;Wnt1-Cre* and *Med23^fx/fx^;Wnt1-Cre* embryos however revealed proper formation of the cranial ganglia in mutant embryos. Scale bars for **(A,B)** is 450 um, **(C,D)** is 375 um.

Our analyses point toward a role for Med23 in regulating WNT/β-catenin signaling during cranial placode development, the perturbation of which results in defects in cranial ganglia formation. However, in addition to placode cells, cranial ganglia also receive a major contribution from another ectodermal cell population, the neural crest. Neural crest cells have been shown to be important for proper cranial placode development and neuronal differentiation (Begbie and Graham, 2001). Because *Med23* is ubiquitously expressed in all cell types, including neural crest cells, we hypothesized that Med23 might also be required in neural crest cells for proper cranial sensory nervous system development, and that the deficiencies observed in cranial placodogenesis in *Med23*^sn/sn^ embryos were a secondary consequence of perturbed neural crest cell development. To test this idea, we generated *Med23*^fx/lx^ animals where exons 12 and 13 of *Med23* were flanked by *loxP* sites. We then conditionally deleted *Med23* specifically in neural crest cell progenitors and their descendants using the *Wnt1-Cre* transgenic line that expresses Cre recombinase in the dorsal neural tube, which encompasses progenitor neural crest cells (Figure 7). *Med23^fx/fx^;Wnt1-Cre* embryos survive until postnatal day (P)0 and exhibit micrognathia (Figures 7A,B). However, these embryos do not display any overt defects in facial prominence, pharyngeal arch or in cranial ganglia development at E10.5 (Figures 7C,D), a time-point when *Med23*^sn/sn^ embryos exhibit severe craniofacial defects, prior to lethality. This is in stark contrast to *Med23*^sn/sn^ embryos, which display considerable diminishment of neurogenesis in the cranial ganglia (Figure 1). Collectively, this suggests that Med23 is not required in neural crest cells for their contribution to cranial ganglia, which supports our original hypothesis that the predominant role for Med23 in cranial sensory nervous system development lies in regulating WNT/β-catenin signaling during cranial placode maturation and neuronal differentiation, the perturbation of which results in defects in cranial ganglia development.

**FIGURE 8:**
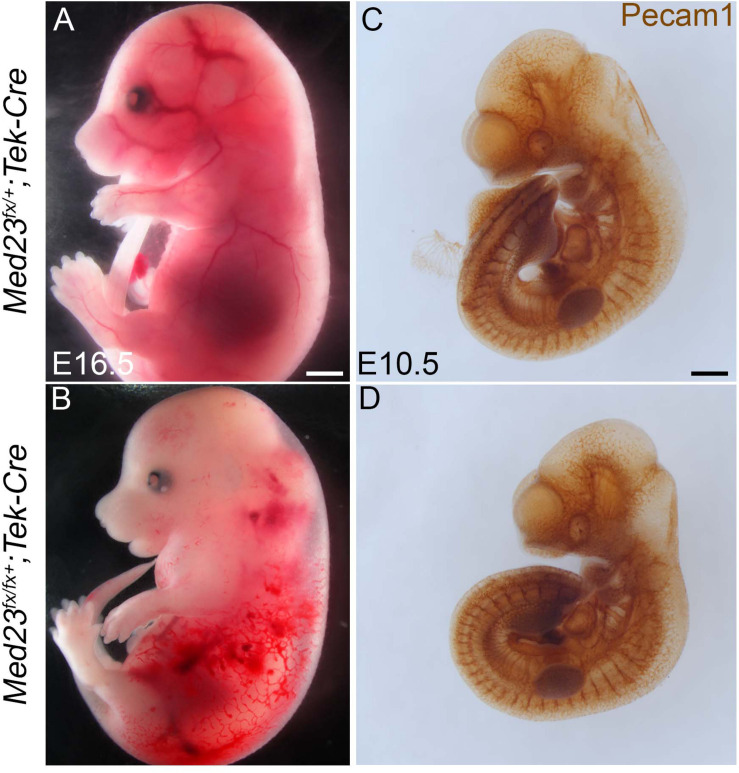
Conditional deletion of Med23 in endothelial cells. **(A,B)** Brightfield images of E16.5 *Med23^fx/+^;Tek-Cre* and *Med23^fx/fx^;Tek-Cre* embryos indicate that the mutant embryos exhibit severe hemorrhaging and edema, probably resulting in lethality. **(C,D)** Immunostaining for endothelial cells using PECAM1 suggests development in *Med23^fx/fx^;Tek-Cre* embryos is normal. Scale bars for **(A,B)** is 450 um, **(C,D)** is 300 um.

Mid-gestation lethality, together with abnormal vascular development and diminished growth are recognizable and consistent features of *Med23*^sn/sn^ embryos relative to control littermates (Figure 1). Because proper vascular network formation and remodeling is critical for embryo survival, we posited that Med23 may be specifically required in endothelial cells for proper neurovascular formation, embryo growth and survival. To test this hypothesis, we conditionally deleted *Med23* in endothelial cells beginning around E8.5 with *Tek-Cre* (*Tie2-Cre*) (Kisanuki et al., 2001; Proctor et al., 2005). *Med23^fx/fx^;Tek-Cre* embryos exhibit embryonic lethality at E16.5 in association with vascular defects including blood hemorrhaging and edema (Figures 8A,B). Surprisingly, *Med23^fx/fx^;Tek-Cre* embryos did not exhibit any obvious gross craniofacial, or vascular defects at E10.5 (Figures 8C,D). Therefore, Med23 is not required in endothelial cells for early embryonic survival. Instead, Med23 appears to be required later in mid-gestation for proper vascular development and maintenance, and also for embryo growth and survival.

### Med23 Is Required at E6.5 for Embryonic Survival and Growth

**FIGURE 9:**
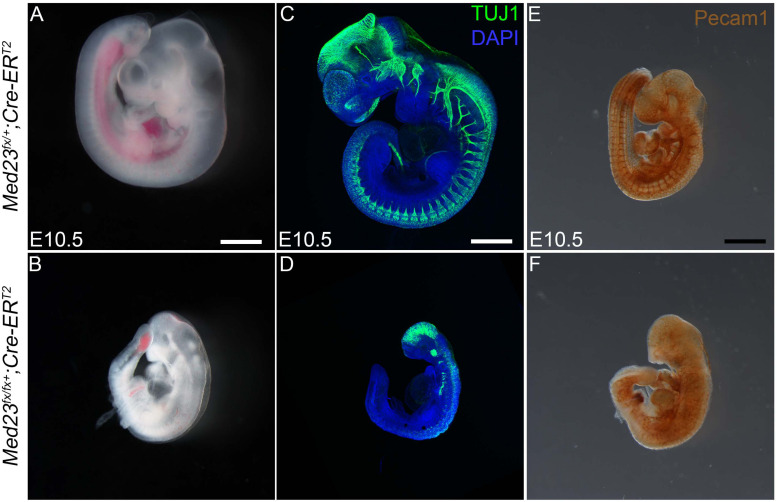
Temporal systemic deletion of Med23 at E6.5 phenocopies *Med23*^sn/sn^ embryos. **(A,B)** *Med2^fx/fx^;Cre-ER^T2^* embryos treated with tamoxifen at E5.5 are small in size compared to *Med2^fx/+^;Cre-ER^T2^* embryos. These embryos also display a shortened frontonasal prominence similar to *Med23*^sn/sn^ embryos. **(C,D)** TuJ1 staining revealed severely hypoplastic cranial as well as vagal ganglia in *Med2^fx/fx^;Cre-ER^T2^* embryos. **(E,F)** PECAM1 staining revealed that endothelial cells are disorganized in the *Med2^fx/fx^;Cre-ER^T2^* embryos compared to control *Med2^fx/+^;Cre-ER^T2^* embryos. Scale bars for **(A,B)** is 250 um, **(C,D)** is 375 um, **(E,F)** is 300 um.

To determine the developmental stage at which Med23 is required for embryo growth and survival, we deleted *Med23* in a temporal manner using *Cre-ER^T2^* transgenic mice in conjunction with tamoxifen induction. Ubiquitous deletion of *Med23* in E8.5 mouse embryos elicits no gross defects in cranial ganglia or vascular development at E10.5, and mutant embryos survive until birth (Supplementary Figure S5). Deletion of *Med23* a day earlier at E7.5 results in embryos that are smaller compared to controls. However, the cranial ganglia and vascular network appear to be properly formed and patterned by E10.5 (Supplementary Figure S6). In contrast tamoxifen gavage at E5.5, leading to *Med23* deletion by E6.5 results in defects in both cranial ganglia as well as vascular development (Figures 9C–F). Furthermore, these embryos die by E10.5 similar to *Med23*^sn/sn^ mutants (Figure 9). This indicates that Med23 is critically required by E6.5 for normal cranial ganglia and vascular network formation and patterning along with mouse embryo growth, development and survival.

## Discussion

Mediator is a multi-protein complex that has been identified in eukaryotic organisms, from yeast to human (Conaway et al., 2005), and which transmits information from transcription factors to RNA polymerase II (Pol II) to regulate transcription. Med23 and some other subunits of the Mediator complex, are however a metazoan innovation (Malik and Roeder, 2000), and their appearance coincides with the evolution of multi-cellular and multi-tissue complex organisms whose embryonic development requires precise control of cell specification, commitment and differentiation.

The cranial ganglia and their associated 12 sensory-motor nerves are vital for proper functioning of the head, face and neck in adult animals. Their development is governed by the reiterated use of key signaling pathways including WNT signaling (Lassiter et al., 2014; Saint-Jeannet and Moody, 2014; Singh and Groves, 2016). In this study, we describe a novel role for the mediator subunit Med23 in craniofacial and cranial ganglia development. More specifically we show that Med23 is essential for embryonic growth and survival and, also that it regulates canonical WNT signaling during cranial placode development, the perturbation of which results in defects in sensory neuron differentiation in the trigeminal and epibranchial ganglia.

Neurons within the trigeminal ganglion are of a dual neural crest or cranial placode origin. In contrast, neurons in the distal epibranchial ganglia are derived from placode cells, while the neural crest cells primarily form glia. Although the origin and development of sensory neurons within the cranial ganglia have been well-described, our understanding of how placodal ectoderm cells are induced to differentiate into sensory neurons during cranial ganglia formation, and the molecular mechanisms that direct this process are not fully understood.

**FIGURE 10:**
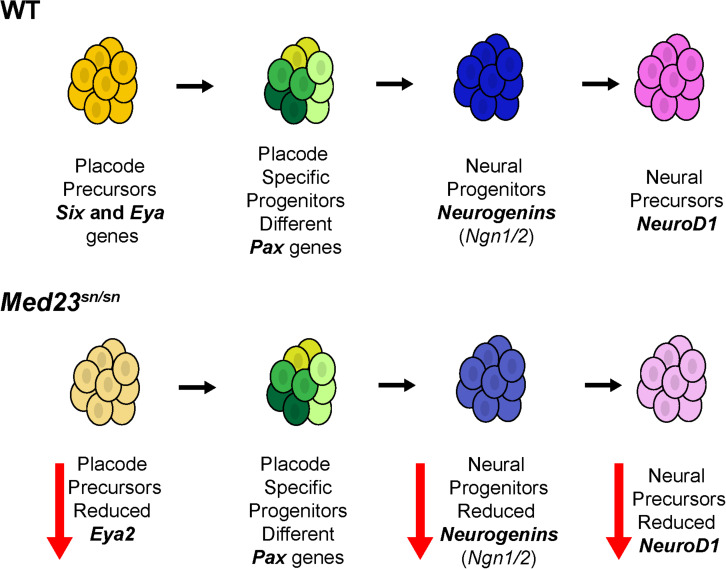
Model for Med23-mediated regulation of cranial placode development. In wild-type embryos, placode precursors express *Six* and *Eya* genes. Their subsequent expression of different *Pax* genes defines them as placode specific progenitor cells. Some of these cells then express *Ngn1* and *Ngn2* thus becoming neural progenitor cells. Ngn1 and Ngn2 regulate the expression of *NeuroD1* which then defines these cells as neural precursors. However, in *Med23*^sn/sn^ embryos, placode precursors exhibit reduced *Eya2* expression, and although Pax genes are similarly expressed in *Med23*^sn/sn^ embryos as they are in wild-type embryos, *Ngn1*, *Ngn2*, and *NeuroD1* are downregulated. Collectively, this reduces the number of neural precursors resulting in defects in cranial ganglia development in *Med23*^sn/sn^ embryos.

In the absence of Med23, *Ngn1, Ngn2*, and *NeuroD1* positive cells are diminished or absent in the trigeminal and epibranchial regions of *Med23^sn/sn^* embryos. This illustrates an important role for Med23 specifically in cranial placode sensory neuron differentiation (Figure 10). Cellular interactions between neural crest cells and placode cells are essential for proper cranial nerve patterning (Shiau et al., 2008; Freter et al., 2013; Theveneau et al., 2013), however, the conditional deletion of *Med23* specifically in neural crest cells did not result in defects in cranial ganglia development in *Med23^fx/fx^;Wnt1-Cre* conditional mutant embryos. This argues against an intrinsic role for Med23 in neural crest cells during their differentiation into trigeminal neurons. However, we cannot completely rule out the possibility that the loss of placode derived neurons within the trigeminal ganglion of *Med23*^sn/sn^ embryos secondarily influences the neurogenic differentiation of neural crest cells that populate the trigeminal ganglion. Nonetheless, our data collectively supports a primary role for Med23 in placode cells during cranial sensory neuron differentiation.

Currently, the role of distinct Mediator subunits in general transcription versus transcription stimulated by specific signaling pathways is poorly understood. However, our transcriptome analyses of *Med23*^sn/sn^ embryos demonstrated that loss of Med23 resulted in an enhancement of WNT signaling. Furthermore, we showed that the regulation of canonical WNT signaling by Med23 is required to initiate and/or maintain the cascade of neurogenic differentiation in the trigeminal and epibranchial placodal regions. Consistent with this observation, we demonstrated that genetically modulating WNT signaling could partially ameliorate the defects in cranial ganglia development in *Med23*^sn/sn^ embryos. Our work therefore has uncovered an important link between the Mediator complex and WNT signaling which integrates the general transcription co-factor machinery with modulation of a major highly conserved signaling pathway important in development, tissue homeostasis and disease.

Interestingly, a similar enhancement in WNT signaling was observed in zebrafish embryos in response to Med10, Med12 or Med13 loss-of-function (Lin et al., 2007). Taken together with our Med23 loss-of-function results, this collectively illustrates the important roles that Mediator and its subunits play in transducing WNT signaling during embryogenesis. However, the role of Med23 and other Mediator subunits, and their effects are context dependent. For example, our results indicate that Med23 promotes cranial placode sensory neuron differentiation through inhibition of WNT signaling. In contrast, Med23 inhibits primitive neural determination of murine embryonic stem cells via the activation of BMP signaling (Zhu et al., 2015). Cross-talk between WNT and BMP signaling has been implicated in many biological events during embryogenesis including neural development, and Wise has been shown to modulate both WNT and BMP signaling (Itasaki et al., 2003). Furthermore, Wise modulates WNT signaling through an interaction with the WNT co-receptor LRP6 (Guidato and Itasaki, 2007; Lintern et al., 2009). This is important because we demonstrated that the defects in cranial sensory neuron differentiation observed in the trigeminal and epibranchial ganglia of *Med23*^sn/sn^ embryos can be ameliorated through combined allelic loss of *Wise* and *Lrp6*.

Wise has also been reported to promote the coalescence of placode cells and neural crest cells during formation and differentiation of the cranial ganglia (Shigetani et al., 2008) and it is interesting to note that despite partial restoration of cranial ganglia sensory neuron differentiation in *Med23^sn/sn^;Wise^+/–^* and *Med23^sn/sn^;Lrp6^+/–^* embryos, the sensory neurons were diffusely and incompletely organized into trigeminal and epibranchial ganglia. Although the defects in cranial ganglia sensory neuron differentiation appear to be primarily associated with perturbation of WNT signaling, we cannot rule out an additional role for Med23 in modulating BMP during cranial placode neurogenesis. However, our transcriptome analyses of *Med23*^sn/sn^ embryos did not reveal mis-regulation of *Bmp4* itself or characteristic downstream targets of BMP signaling.

A key question that emerges from our work is how a ubiquitously expressed transcriptional co-factor subunit can play tissue specific differentiation roles during embryogenesis. A number of Mediator subunits have previously been shown to exhibit tissue specific roles or effects (Yin and Wang, 2014), and one possibility is that the composition of the Mediator complex may be quite dynamic and comprise a different number and combination of subunits at different times and in different tissues during embryogenesis and adult homeostasis. This hypothesis assumes that the subunits that are required for core Mediator functionality (e.g., Pol II binding) are always present within the complex, while others, that perhaps exist in the tail of Mediator such as Med23, are only present at specific times or in specific tissues. *Med23* loss-of-function in mouse embryos does not disrupt preimplantation development or germ layer specification during gastrulation, when WNT signaling through the Lrp5 and Lrp6 co-receptors is known to be required (Kelly et al., 2004). Hence Med23 does not uniformly impact levels of WNT signaling in all contexts where it is expressed. It is only around E9.5 that developmental anomalies become apparent in *Med23*^sn/sn^ embryos. We propose therefore that Med23 may be dynamically required for the regulation of specific genes in specific cells and that there is some degree of context-dependence in its ability to exert its regulatory activity. In support of this idea, the response of transcription factors such as Egr1 to *Med23* loss-of-function is different in embryonic stem cells versus fibroblasts (Balamotis et al., 2009).

A precedent for the differential and dynamic role of Med23 exists with respect to adipocyte and smooth muscle cells (Yin et al., 2012), in osteoblast differentiation and bone development (Liu et al., 2016), in melanocytes (Xia et al., 2017) and in invariant natural killer T cells (Xu et al., 2018). More specifically, Med23 has been shown to directly interact with the ETS1 family member, phospho-Elk1, which is central to the transcriptional activation of *Egr2* during adipocyte differentiation (Wang et al., 2009). In addition, MED23 favors ELK1-SRF binding to smooth muscle cell gene promoters to repress gene activity, whereas the absence of MED23 promotes MAL-SRF binding to smooth muscle cell gene promoters resulting in gene activation. Med23 also binds to Runx2 and co-operates with Runx2 to promote osteoblast differentiation (Liu et al., 2016). Lastly, Med23 loss-of-function in thymocytes blocks invariant natural killer cell development by regulating the expression of c-Jun (Xu et al., 2018). Taken together with our data, this highlights the context-dependent nature and importance of Med 23 – transcription factor interactions during development.

Our analysis of Med23 expression during murine embryogenesis revealed that Med23 is ubiquitously expressed (Figure 2). This combined with the relatively late onset of a developmental phenotype in *Med23*^sn/sn^ embryos at around E9.5, is suggestive of functional redundancy among the Mediator subunits, and interestingly, knockouts of currently known Med23 binding partners do not recapitulate the phenotype of *Med23*^sn/sn^ embryos (Screpanti et al., 1996; Cesari et al., 2004; Oqani et al., 2016). This implies the existence of other as yet unknown Med23 binding partners, the interactions of which are likely crucial for eliciting Med23’s role in regulating WNT signaling during sensory neuron differentiation in the trigeminal and epibranchial placodes.

Med23 has been shown to be important for gene expression and tissue development in *C. elegans, Drosophila*, zebrafish and mouse embryos (Singh and Han, 1995; Stevens et al., 2002; Kim and Lis, 2005; Wang et al., 2005, 2009; Yin and Wang, 2014; Liu et al., 2016), and interestingly, mutations in *MED23* in humans have been linked to intellectual disability, microcephaly and cardiovascular anomalies together with overall growth retardation defects (Lüneburg et al., 2014; Lionel et al., 2016; Hashemi-Gorji et al., 2019). Our work on *Med23*^sn/sn^ embryos has uncovered a previously unexplored role for Med23-containing Mediator in craniofacial and cranial ganglia development and it is tempting to speculate that a similar effect on WNT signaling and neuronal development may contribute to the pathogenesis of the neurological phenotypes observed in humans.

Protein binding studies have shown that the Mediator complex, through its various subunit interactions, acts as a hub for integrating cellular signaling cascades, transcription factors and the RNA Pol II machinery (Poss et al., 2013; Yin and Wang, 2014). Mediator regulates various transcriptional processes, including, but not limited to, the assembly of the pre-initiation complex (PIC) at the transcription initiation site, transcript elongation and termination, mRNA processing, and chromatin architecture. Furthermore, the differential interaction of specific mediator subunits with distinct transcription factors is critical to Mediator function. Potentially, a single transcription factor or activator can interact with multiple Mediator-binding sites and thus activate transcription from the same promoter differently in distinct cell types depending on which contacts are made. For example, the transcriptional activator Gcn4 binds to a Mediator subcomplex consisting of MED2, MED3, and MED15 (Park et al., 2000; Zhang et al., 2004). In contrast, the mammalian glucocorticoid receptor interacts with a distinct set of Mediator subunits including MED1 (Chen and Roeder, 2007), MED14 (Chen et al., 2006), and MED15 (Kim et al., 2008). Similar differential and dynamic roles for other Mediator subunits such as Med26 or the Mediator associated sub-complex CDK8, have also been reported (Yin and Wang, 2014). As might be expected from its central role in transcription regulation, Mediator and its subunits are crucial for gene expression and cellular differentiation during embryonic development. Consequently, it will be interesting in the future to determine the stoichiometric composition of subunits of Mediator, together with their compete repertoire of subunit-transcription factor interactions as well DNA-binding capabilities within various cell types and tissues and at different times, to understand their relative importance in embryo development and adult homeostasis.

## Data Availability Statement

The datasets generated for this study can be found in the GEO Series, accession number GSE144327.

## Ethics Statement

The animal study was reviewed and approved by the Stowers Institute of Medical Research (SIMR) IACUC protocols (2019-094 and 2019-097).

## Author Contributions

All authors listed have made a substantial, direct and intellectual contribution to the work, and approved it for publication.

## Conflict of Interest

The authors declare that the research was conducted in the absence of any commercial or financial relationships that could be construed as a potential conflict of interest.

## Footnotes

1 https://microarray.org/sfgf/meebo.do

2 https://www.ncbi.nlm.nih.gov/geo/query/acc.cgi?acc=GSE144327

## Supplementary Material

Supplementary Figure 1 *Med23*^bgeo/bgeo^ embryos exhibit developmental delay. **(A)** Generation of the *Med23*^bgeo^ allele **(B,C)** DAPI stained E9.5 control and *Med23*^bgeo/bgeo^ littermate embryos reveal developmental delay in *Med23*^bgeo/bgeo^ embryos, in concert with a failure to undergo axial turning. Scale bar is 100 um.

Supplementary Figure 2 Complementation cross indicates *snouty* is a null allele of *Med23*. Wild-type **(A,C,E)** and *Med23*^bgeo/sn^ **(B,D,F)** littermate embryos stained with DAPI **(A,B)**, immunostained with TuJ1 **(C,D)**, or PECAM1 **(E,F)**. Morphological, neural and vascular defects in *Med23*^bgeo/sn^ embryos are similar to *Med23*^sn/sn^ embryos suggesting that *snouty* is likely a null allele of *Med23*. Scale bar is 100 um.

Supplementary Figure 3 Placental development is not affected in *Med23*^sn/sn^ embryos. **(A–D)** Wild-type and *Med23*^sn/sn^ placentas sectioned along two separate planes and stained with hematoxylin and eosin shows that the trophoblast and allantois layers are appropriately formed in both wild-type and *Med23*^sn/sn^ placenta. The dotted line denotes the separation of the maternal decidua to the placenta. MD, maternal deciduas; T, trophoblast; AL, allantois. Scale bar for **(A,C)** is 200 um and **(B–D)** and 150 um.

Supplementary Figure 4 Peripheral nervous system formation is not affected by loss of one copy of *Lrp6* or *Wise*. **(A–C)** TuJ1 immunostaining of E9.5 *Lrp6*^+/–^ and *Wise*^+/–^ embryos revealed proper formation of the cranial and trunk peripheral nervous systems, as well as midbrain neuron differentiation. **(D,E)** Intercrossing the *Lrp6*^lacZ^ mouse line demonstrated that E9.5 wild-type and *Med23*^sn/sn^ embryos display similar *Lrp6* expression as evidenced by X-gal staining. **(F,G)** Intercrossing the *Wise*^lacZ^ mouse line demonstrated that wild-type and *Med23*^sn/sn^ embryos display similar patterns of *Wise* activity as evidenced by X-gal staining. Scale bar is 300 um.

Supplementary Figure 5 Temporal systemic deletion of Med23 at E8.5 does not affect cranial ganglia development. **(A,B)** *Med2^fx/fx^;Cre-ER^T2^* embryos treated with tamoxifen at E7.5 survive until P0 with no discernable defects. **(C,D)** TuJ1 staining indicates proper patterning of cranial ganglia in *Med2^fx/fx^;Cre-ER^T2^* embryos. **(E,F)** PECAM1 staining reveals that endothelial cells are properly formed and organized into networks in *Med2^fx/fx^;Cre-ER^T2^* embryos compared to *Med2^fx/+^;Cre-ER^T2^* controls. Scale bar for **(A,B)** is 500 um, **(C,D)** is 375 um, **(C–F)** is 300 um.

Supplementary Figure 6 Temporal systemic deletion of Med23 at E7.5 leads to a mild cranial ganglia patterning defects. **(A,B)** *Med2^fx/fx^;Cre-ER^T2^* embryos treated with tamoxifen at E6.5 survive until E15.5. However, at E14.5, they are smaller in size compared to *Med2^fx/+^;Cre-ER^T2^* embryos. **(C,D)** TuJ1 staining revealed mild patterning defects in the cranial ganglia of *Med2^fx/fx^;Cre-ER^T2^* embryos. **(E,F)** PECAM1 staining showed that endothelial cells are unaffected in *Med2^fx/fx^;Cre-ER^T2^* embryos compared to *Med2^fx/+^;Cre-ER^T2^* controls. Scale bar for **(A,B)** is 500 um, **(C,D)** is 375 um, **(C–F)** is 300 um.

Supplementary Figure 7 Pathway analysis of genes differentially regulated in *Med23*^sn/sn^ embryos compared to wild-type. Cytoscape network plot of results from Ingenuity Pathway Analysis illustrating the association of the canonical WNT/β-catenin signaling pathway with the differential expression of multiple genes in *Med23*^sn/sn^ embryos compared to controls.

Supplementary Table 1 Examples of genes with downregulated expression in *Med23*^sn/sn^ embryos compared to wild-type littermate controls.

Supplementary Table 2 List of genes differentially regulated in *Med23*^sn/sn^ embryos compared to wild-type with *p* < 0.01.
