# Supplementary figures and images for: The Mediator Subunit, Med23 Is Required for Embryonic Survival and Regulation of Canonical WNT Signaling During Cranial Ganglia Development
Source: Front Physiol. 2020 Oct 22;11:531933. doi: 10.3389/fphys.2020.531933 (PMC7642510; doi:10.3389/fphys.2020.531933)

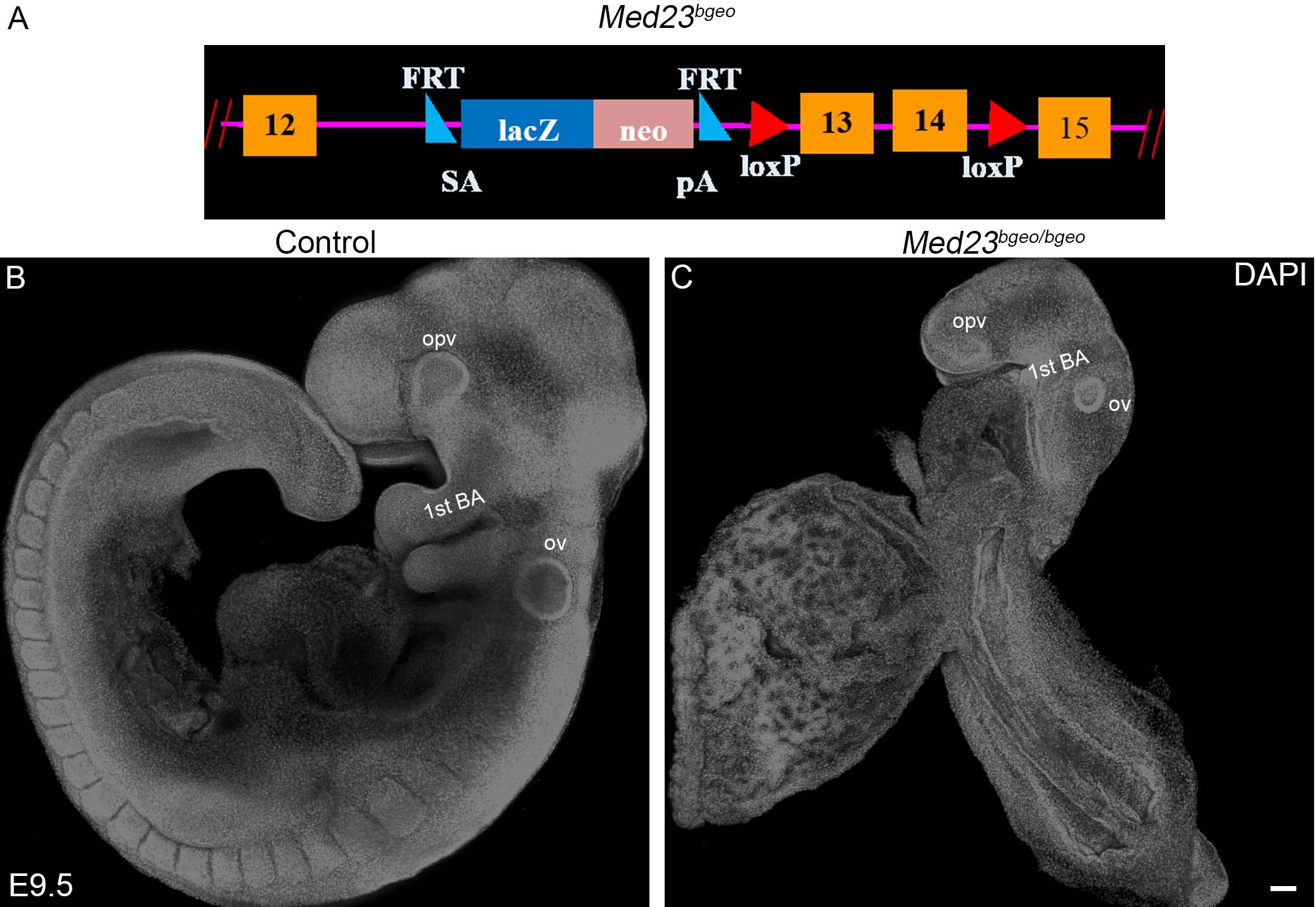

Supplement: Supplementary Figure 1 — Med23bgeo/bgeo embryos exhibit developmental delay. (A) Generation of the Med23bgeo allele (B,C) DAPI stained E9.5 control and Med23bgeo/bgeo littermate embryos reveal developmental delay in Med23bgeo/bgeo embryos, in concert with a failure to undergo axial turning. Scale bar is 100 um. [file Image_1.JPEG]

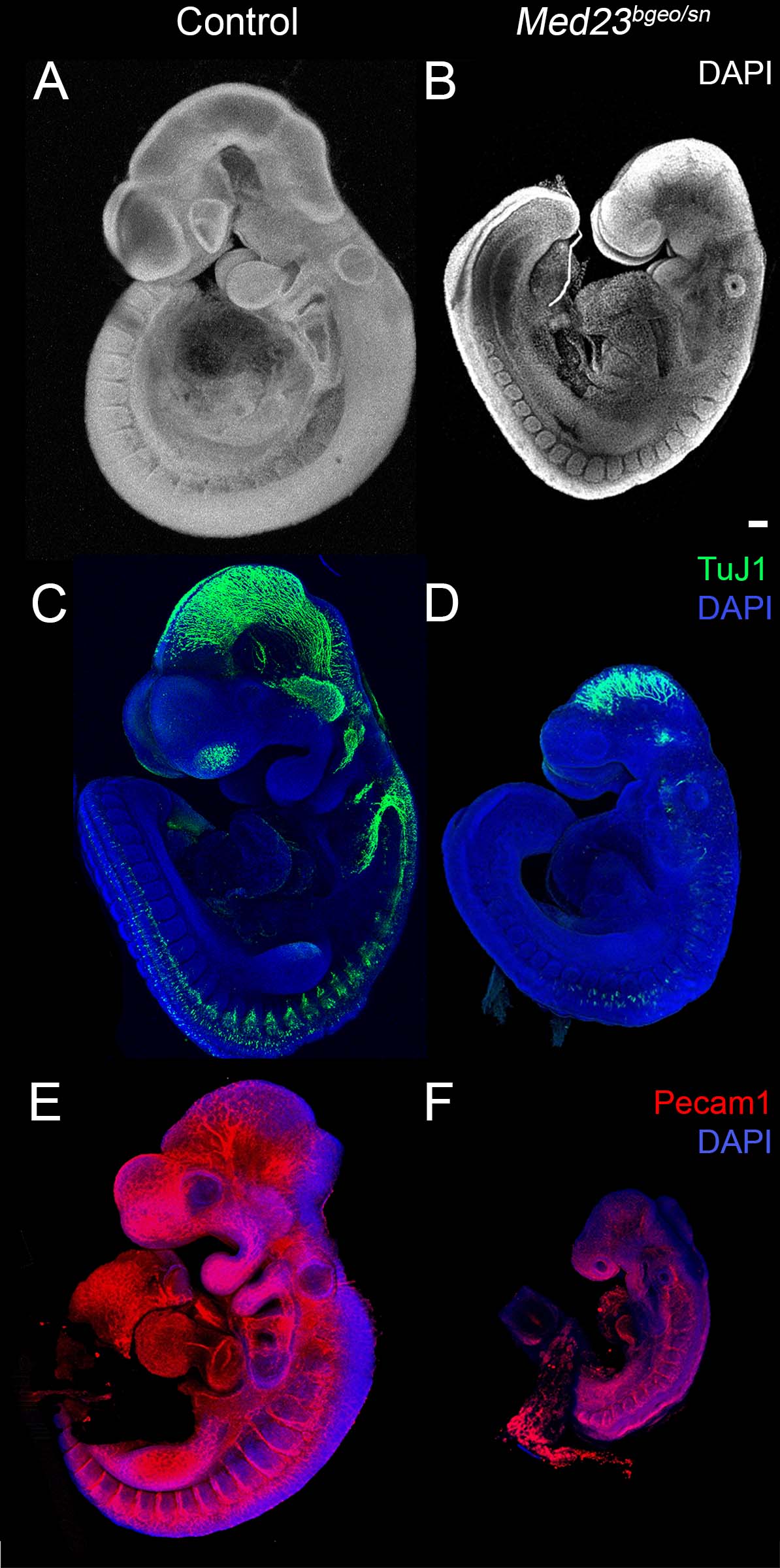

Supplement: Supplementary Figure 2 — Complementation cross indicates snouty is a null allele of Med23. Wild-type (A,C,E) and Med23bgeo/sn (B,D,F) littermate embryos stained with DAPI (A,B), immunostained with TuJ1 (C,D), or PECAM1 (E,F). Morphological, neural and vascular defects in Med23bgeo/sn embryos are similar to Med23sn/sn embryos suggesting that snouty is likely a null allele of Med23. Scale bar is 100 um. [file Image_2.JPEG]

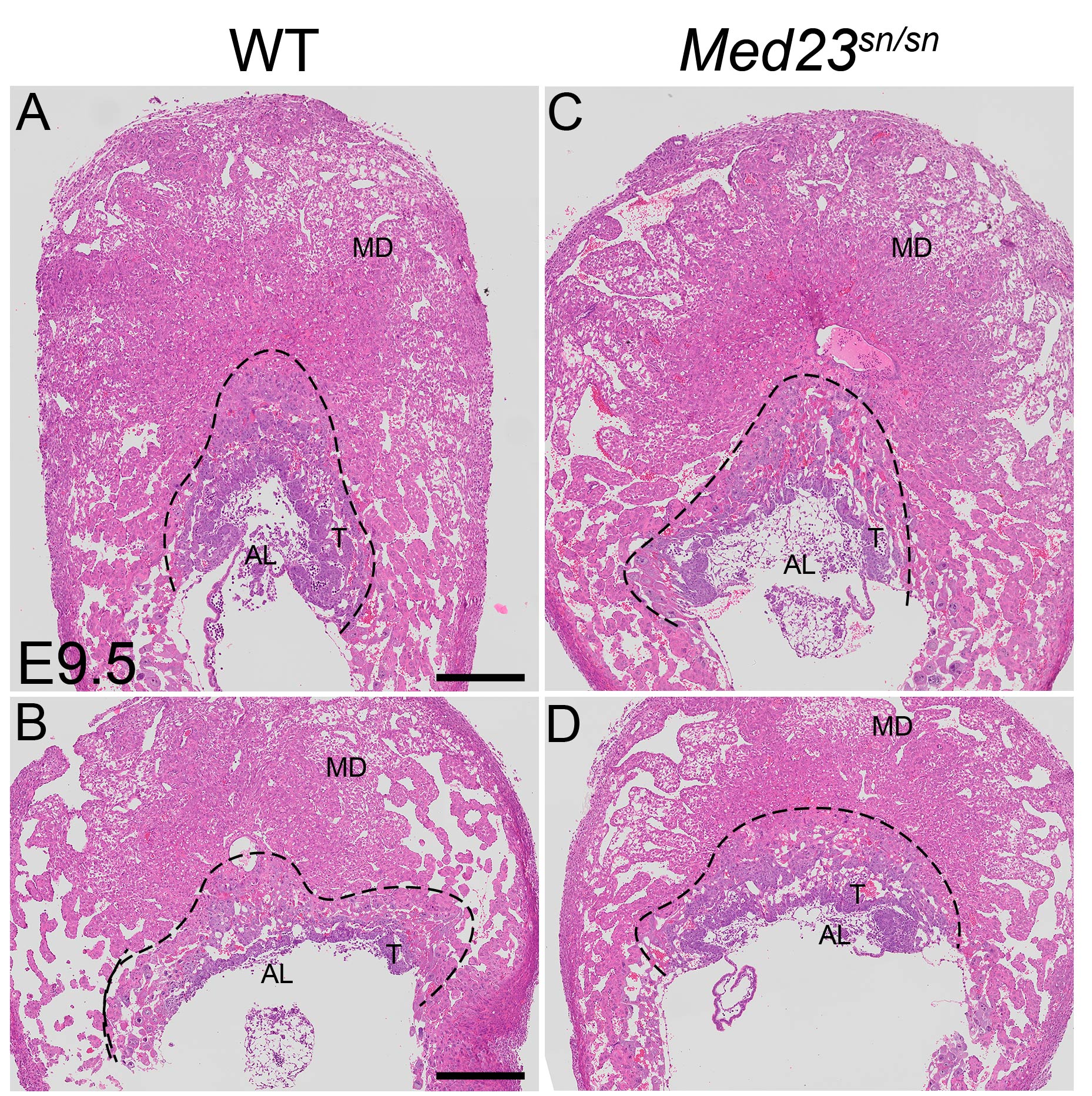

Supplement: Supplementary Figure 3 — Placental development is not affected in Med23sn/sn embryos. (A–D) Wild-type and Med23sn/sn placentas sectioned along two separate planes and stained with hematoxylin and eosin shows that the trophoblast and allantois layers are appropriately formed in both wild-type and Med23sn/sn placenta. The dotted line denotes the separation of the maternal decidua to the placenta. MD, maternal deciduas; T, trophoblast; AL, allantois. Scale bar for (A,C) is 200 um and (B–D) and 150 um. [file Image_3.JPEG]

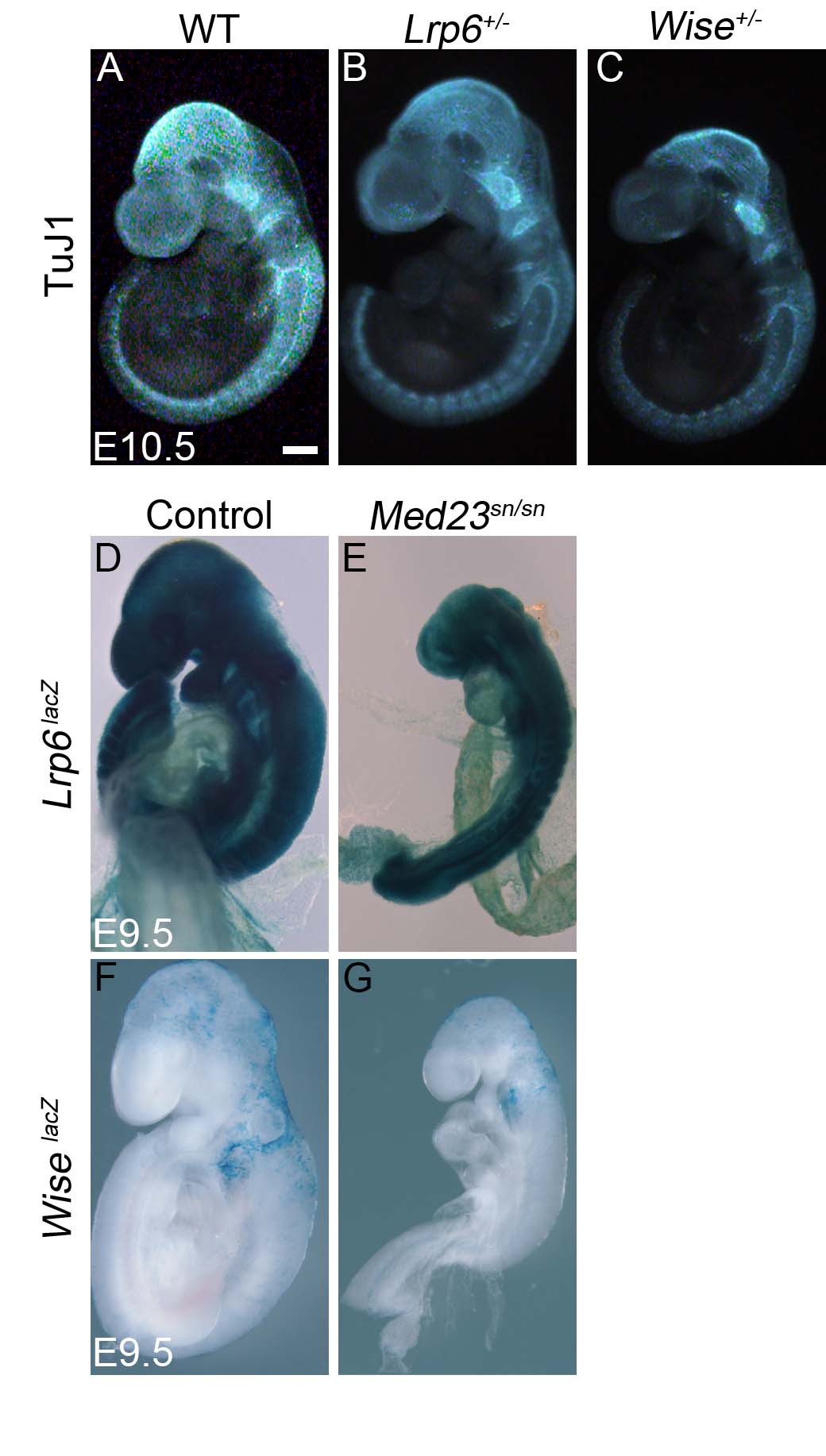

Supplement: Supplementary Figure 4 — Peripheral nervous system formation is not affected by loss of one copy of Lrp6 or Wise. (A–C) TuJ1 immunostaining of E9.5 Lrp6+/– and Wise+/– embryos revealed proper formation of the cranial and trunk peripheral nervous systems, as well as midbrain neuron differentiation. (D,E) Intercrossing the Lrp6lacZ mouse line demonstrated that E9.5 wild-type and Med23sn/sn embryos display similar Lrp6 expression as evidenced by X-gal staining. (F,G) Intercrossing the WiselacZ mouse line demonstrated that wild-type and Med23sn/sn embryos display similar patterns of Wise activity as evidenced by X-gal staining. Scale bar is 300 um. [file Image_4.JPEG]

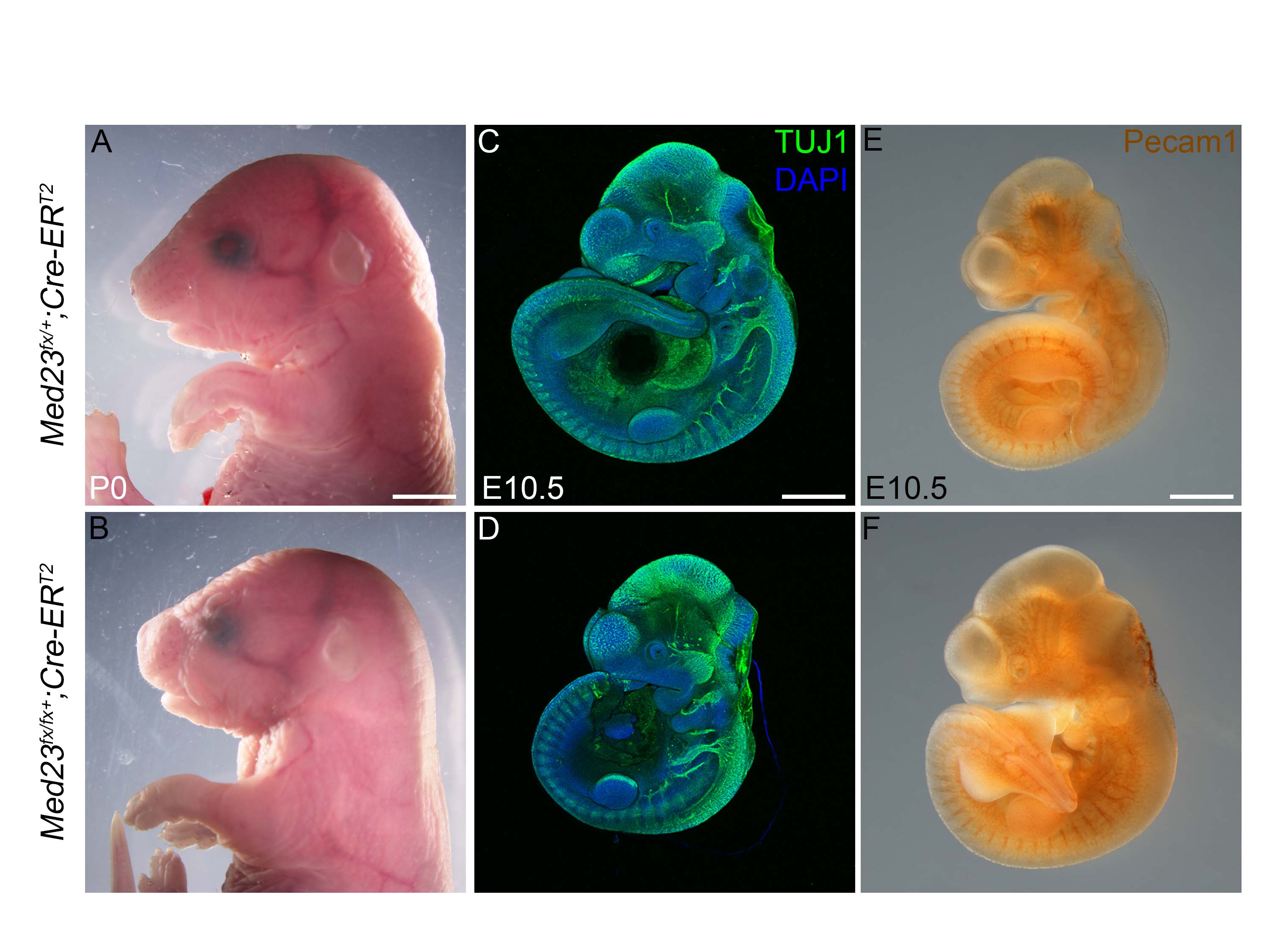

Supplement: Supplementary Figure 5 — Temporal systemic deletion of Med23 at E8.5 does not affect cranial ganglia development. (A,B) Med2fx/fx;Cre-ERT2 embryos treated with tamoxifen at E7.5 survive until P0 with no discernable defects. (C,D) TuJ1 staining indicates proper patterning of cranial ganglia in Med2fx/fx;Cre-ERT2 embryos. (E,F) PECAM1 staining reveals that endothelial cells are properly formed and organized into networks in Med2fx/fx;Cre-ERT2 embryos compared to Med2fx/+;Cre-ERT2 controls. Scale bar for (A,B) is 500 um, (C,D) is 375 um, (C–F) is 300 um. [file Image_5.JPEG]

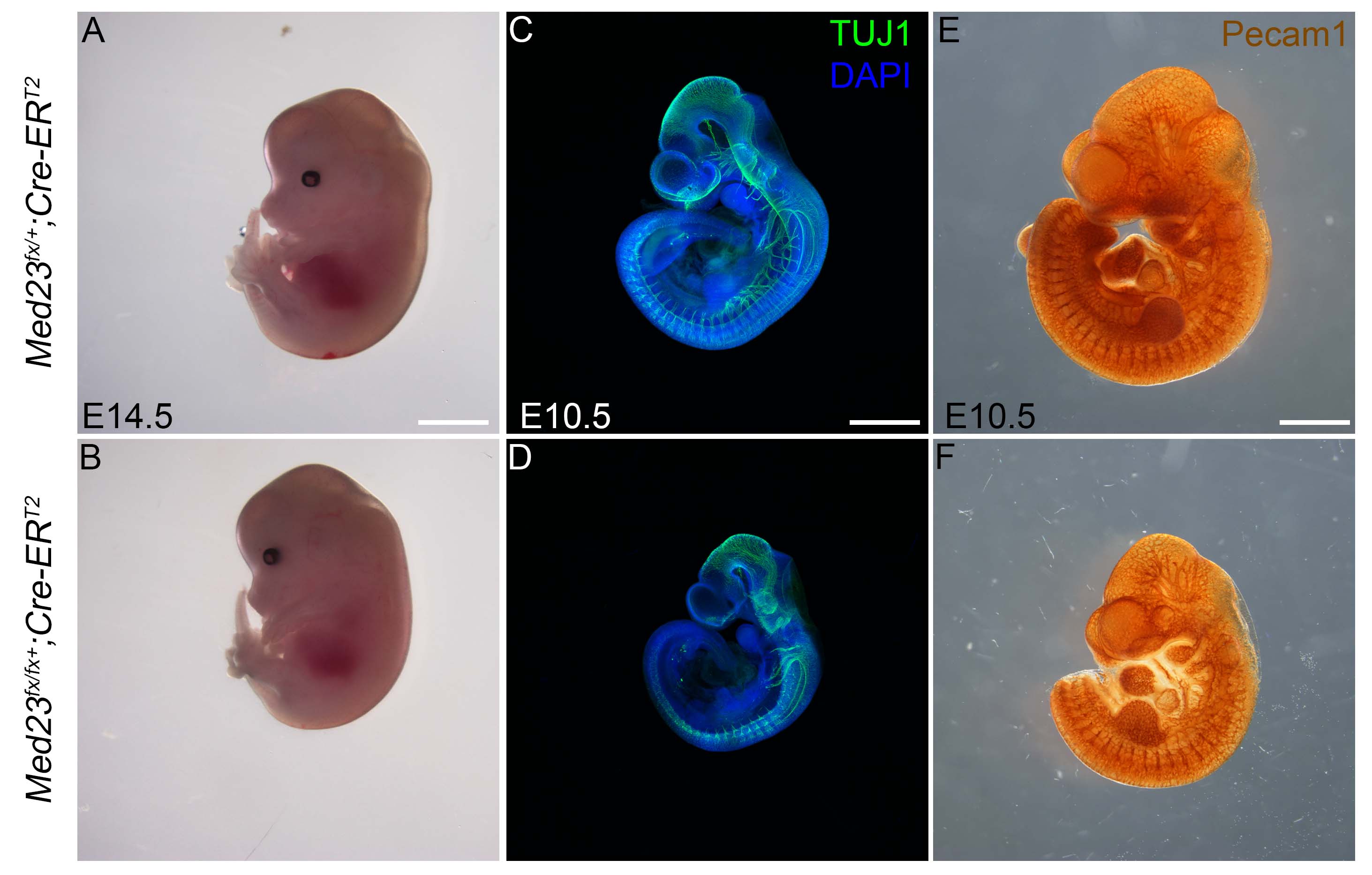

Supplement: Supplementary Figure 6 — Temporal systemic deletion of Med23 at E7.5 leads to a mild cranial ganglia patterning defects. (A,B) Med2fx/fx;Cre-ERT2 embryos treated with tamoxifen at E6.5 survive until E15.5. However, at E14.5, they are smaller in size compared to Med2fx/+;Cre-ERT2 embryos. (C,D) TuJ1 staining revealed mild patterning defects in the cranial ganglia of Med2fx/fx;Cre-ERT2 embryos. (E,F) PECAM1 staining showed that endothelial cells are unaffected in Med2fx/fx;Cre-ERT2 embryos compared to Med2fx/+;Cre-ERT2 controls. Scale bar for (A,B) is 500 um, (C,D) is 375 um, (C–F) is 300 um. [file Image_6.JPEG]

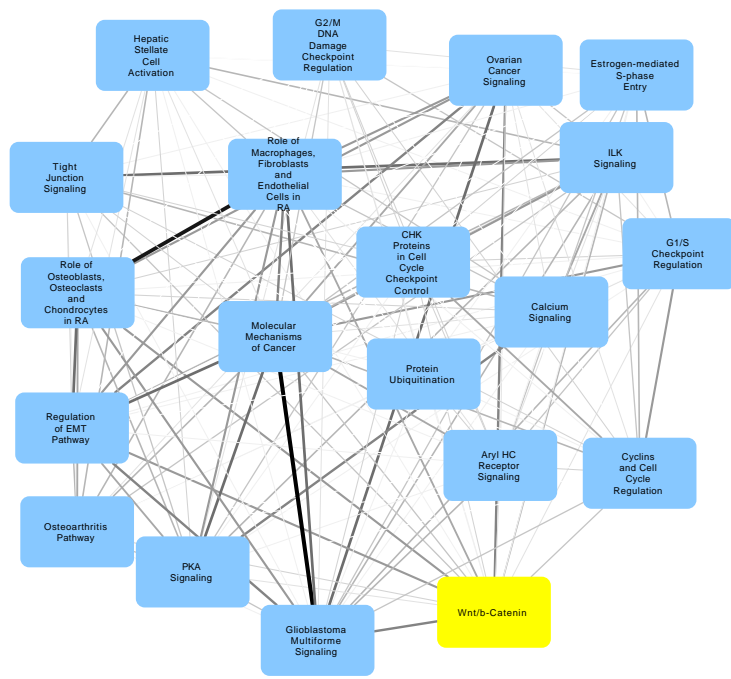

Supplement: Supplementary Figure 7 — Pathway analysis of genes differentially regulated in Med23sn/sn embryos compared to wild-type. Cytoscape network plot of results from Ingenuity Pathway Analysis illustrating the association of the canonical WNT/β-catenin signaling pathway with the differential expression of multiple genes in Med23sn/sn embryos compared to controls. [file Image_7.pdf]
