# Supplementary material for: The Mediator Subunit, Med23 Is Required for Embryonic Survival and Regulation of Canonical WNT Signaling During Cranial Ganglia Development
Source: Front Physiol. 2020 Oct 22;11:531933. doi: 10.3389/fphys.2020.531933 (PMC7642510; doi:10.3389/fphys.2020.531933)
Supplement: Supplementary Table 2 — List of genes differentially regulated in Med23sn/sn embryos compared to wild-type with p < 0.01. [file Table_2.pdf]

Symbol	Gene Name	log2FC (Mt vs WT)	Fold change (Mt vs WT)
Egr1	Early growth response 1	-3.25	0.11
Raet1c	Retinoic acid early transcript gamma	-1.49	0.36
Irak3	Interleukin-1 receptor-associated kinase 3	-1.10	0.46
**Dkk1**	**Dickkopf homolog 1**	**-0.95**	**0.52**
Hemgn	Hemogen	-0.94	0.52
Mab21l1	Mab-21-like 1 (C. elegans)	-0.92	0.53
Nkx6-2	NK6 transcription factor related, locus 2 (Drosophila)	-0.75	0.59
Tbx1	T-box 1	-0.72	0.61
**Neurod1**	**Neurogenic differentiation 1**	**-0.69**	**0.62**
Dlk1	Delta-like 1 homolog (Drosophila)	-0.67	0.63
**Ccnd1**	**Cyclin D1**	**-0.64**	**0.64**
